# Patterns in the Prevalence of Unvaccinated Children Across 36 States and Union Territories in India, 1993-2021

**DOI:** 10.1001/jamanetworkopen.2022.54919

**Published:** 2023-02-10

**Authors:** Sunil Rajpal, Akhil Kumar, Mira Johri, Rockli Kim, S. V. Subramanian

**Affiliations:** 1 Interdisciplinary Program in Precision Public Health, Department of Public Health Sciences, Graduate School of Korea University, Seoul, South Korea; 2 Department of Economics, FLAME University, Pune, India; 3 Turner Fenton Secondary School, Brampton, Ontario, Canada; 4 Center for Geographic Analysis, Harvard University, Cambridge, Massachusetts; 5 Centre de Recherche du Centre Hospitalier, University of Montréal, Montréal, Québec, Canada; 6 Department of Management, Evaluation, and Health Policy, School of Public Health, University of Montréal, Montréal, Québec, Canada; 7 Division of Health Policy and Management, College of Health Science, Korea University, Seoul, South Korea; 8 Harvard Center for Population and Development Studies, Cambridge, Massachusetts.; 9 Department of Social and Behavioral Sciences, Harvard T.H. Chan School of Public Health, Boston, Massachusetts

## Abstract

**Importance:**

Children who do not receive any routine vaccinations (ie, who have 0-dose status) are at elevated risk of death, morbidity, and socioeconomic vulnerabilities that limit their development over the life course. India has the world’s highest number of children with 0-dose status; analysis of national and subnational patterns is the first important step to addressing this problem.

**Objectives:**

To examine the patterns among children with 0-dose immunization status across all 36 states and union territories (UTs) in India over 29 years, from 1993 to 2021, and to elucidate the relative share of multiple geographic regions in the total geographic variation in 0-dose immunization.

**Design, Setting, and Participants:**

This repeated cross-sectional study analyzed all 5 rounds of India’s National Family Health Survey (1992-1993, 1998-1999, 2005-2006, 2015-2016, and 2019-2021) to compare the prevalence of children with 0-dose status across time-space and geographic regions. The Integrated Public Use of Microdata Series was used to construct comparable geographic boundaries for states and UTs across surveys. The study included a total of 125 619 live children aged 12 to 23 months who were born to participating women.

**Main Outcomes and Measures:**

The outcome was a binary indicator of children’s 0-dose vaccination status, coded as children aged 12 to 23 months at the time of the survey who had not received the first dose of the diphtheria-tetanus-pertussis–containing vaccine. The significance of each geographic unit was computed using the variance partition coefficient (VPC).

**Results:**

Among 125 619 children, the national prevalence of those with 0-dose status in India decreased from 33.4% (95% CI, 32.5%-34.2%) in 1993 to 6.6% (95% CI, 6.4%-6.8%) in 2021. A substantial reduction in the IQR of 0-dose prevalence across states from 30.1% in 1993 to 3.1% in 2021 suggested a convergence in state disparities. The prevalence in the northeastern states of Meghalaya (17.0%), Nagaland (16.1%), Mizoram (14.3%), and Arunachal Pradesh (12.6%) remained relatively high in 2021. Prevalence increased between 2016 and 2021 in 10 states, including several traditionally high-performing states and UTs, such as Telangana (1.16 percentage points) and Sikkim (0.92 percentage points). In 2021, 53.0% of children with 0-dose status resided in the populous states of Uttar Pradesh, Bihar, and Maharashtra. A multilevel analysis comparing the share of variation at the state, district, and cluster (primary sampling unit) levels revealed that clusters accounted for the highest share of the total variation in 2016 (44.7%; VPC [SE], 1.04 [0.32]) and 2021 (64.3%; VPC [SE], 0.38 [0.12]).

**Conclusions and Relevance:**

In this cross-sectional study, findings from approximately 3 decades of analysis suggest the need for sustained efforts to target populous states like Uttar Pradesh and Bihar and northeastern parts of India. The resurgence of 0-dose prevalence in 10 states highlights the importance of programs like Intensified Mission Indradhanush 4.0, a major national initiative to improve immunization coverage. Prioritizing small administrative units will be important to strengthening India’s efforts to bring every child into the immunization regime.

## Key Points

**Question:**

What are the patterns among children who missed the first dose of the diphtheria-tetanus-pertussis vaccine in India from 1993 to 2021?

**Findings:**

In this cross-sectional study of 125 619 children aged 12 to 23 months in India, the prevalence of children who did not receive a single dose of the diphtheria-tetanus-pertussis vaccine decreased from 33.4% to 6.6% between 1993 and 2021, with Uttar Pradesh, Bihar, and Maharashtra accounting for 53.0% of the burden. While interstate inequalities converged notably toward lower prevalence levels, states like Punjab, Telangana, and Andhra Pradesh experienced an increase in prevalence from 2016 to 2021.

**Meaning:**

These findings suggest that sustained efforts are warranted to target high-burden states in India; prioritizing small administrative units will be important to achieving the Immunization Agenda 2030 goals endorsed by the World Health Organization.

## Teaser

This cross-sectional study uses data from National Family Health Surveys to examine patterns among children aged 12 to 23 months who have not received a single dose of the diphtheria-tetanus-pertussis vaccine across all states and union territories in India from 1993 to 2021.

## Introduction

Timely immunization is an important intervention to improve survival outcomes among children. Over 2 decades, low- and middle-income countries have achieved notable improvements in vaccination coverage, inducing substantial mortality reductions.^1^ However, in 2019, approximately 14.5 million infants globally did not receive a single vaccine dose against diphtheria, tetanus, or pertussis (DTP).^2,3^ Lack of receipt of the first injection of the DTP-containing vaccine (DTP1) in the first year of life, referred to as 0-dose status, signals a lack of access to routine immunization as a whole.^4,5^ Immunization Agenda 2030 (IA2030), endorsed by the World Health Organization in May 2021, aims to increase the reach of routine immunizations and halve the prevalence of children with 0-dose status by 2030.^6^

The global count of children with 0-dose status was estimated at 18 million in 2021, an increase associated with COVID-19 pandemic–induced disruptions that have affected the vaccine delivery system worldwide,^7,8^ with an especially severe impact in South Asia.^9^ Although more than three-fourths of children aged 12 to 23 months in India are fully vaccinated,^10^ more than 3 million children were unable to receive any routine vaccinations in 2021, making India the country with the highest number of children with 0-dose status worldwide.^9^ Besides being vulnerable to vaccine-preventable diseases,^1^ children with 0-dose status are also at higher risk of poor health and nutritional outcomes, leading to a high risk of morbidity and poor growth trajectories over the life course.^11,12^ Children with 0-dose immunization status also represent an important marker of general vulnerability, especially in low- and middle-income countries like India.^11^ A higher concentration of children with 0-dose status among low-income and marginalized populations has been observed in previous studies.^5,6,13^ Added disadvantages pertaining to geographic,^14^ religious,^11^ political, gender,^15^ and health care system^16^ factors also play a role in this inequality.^5^ More specifically, children born in rural and Muslim households in India and children of mothers with low educational levels were more likely to belong to the 0-dose group.^8,11^ Collectively, existing literature^11^ points toward the cumulative consequences of multiple sources of deprivation in the form of children with 0-dose status. Therefore, persistent evidence of a population of children with 0-dose status suggests a need for more timely vaccine interventions along with a successful targeting strategy to reshape lifelong opportunities among millions of children in the country.

India has notably achieved self-sufficiency in vaccine production, improvised cold chain and logistics management, and strengthened reporting of vaccine-preventable diseases, thereby representing substantial progress in immunization coverage.^11^ Recent programs like Mission Indradhanush (MI) in 2014 and its intensified versions in 2017 (Intensified Mission Indradhanush [IMI] 2.0) and 2022 (IMI 4.0), have furthered such efforts with a targeted approach at the district level.^17^ It may be noted that the success of such initiatives warrants a focus on bringing children with 0-dose status into the immunization regimen at the state and local levels. Examining patterns in the geographic clustering of children with 0-dose status is the first important step to addressing the details of the problem. Given the large count of children with 0-dose status in India with widespread spatial heterogeneities,^17^ examining patterns in regional inequalities and high-burden areas can offer valuable insights, thereby delineating both low- and high-performing states.

With this aim, we analyzed 29 years of data (1993-2021) to discern the patterns and progress over time in reaching children with 0-dose status across all 36 states and union territories (UTs) in India. Specifically, we used microdata from 5 rounds of a cross-sectional survey to discern comparable state-specific information on children with 0-dose status across time and space. This process included a recalibration of geographic identifiers (clusters and districts) to allow estimations for newly formed states and UTs between 1993 and 2021. We also conducted multilevel analysis to elucidate the relative share of multiple geographic regions (clusters, districts, and states) in the total variation among children with 0-dose status. For the most recent survey round, the study also aimed to offer insight into the absolute burden of children with 0-dose status across states and UTs. To our knowledge, this repeated cross-sectional study is the first to identify comparable state-level patterns in children with 0-dose status in India over approximately 3 decades.

## Methods

### Study Design and Participants

We used publicly available anonymized data from all 5 cross-sectional rounds of India’s National Family Health Survey (NFHS), covering a maximum time frame of 29 years, between 1992 and 2021.^10,18,19,20,21^ After giving written informed consent, primary caregivers (typically mothers) provided information on the vaccination status of their children. The NFHS of India adopts a 2-stage stratified random sampling design (eAppendix 1 in Supplement 1).^10,18,19,20,21^ Cross-sectional analytic samples were constructed for each of the 5 rounds, and the final sample included all children aged 12 to 23 months born to the participating women; dead children and those with missing information on geographic location or vaccination status were excluded.^10,18,19,20,21^ The construction of the analytic sample is shown in eFigure 1 in Supplement 1. The institutional review board of the Harvard Medical School Longwood Campus determined that research ethics review was not required for this study because it relied exclusively on publicly accessible secondary use of anonymized information from the Demographic and Health Surveys (DHS) program.^22^ This study followed Strengthening the Reporting of Observational Studies in Epidemiology (STROBE) guideline for cross-sectional studies.

### Outcome

The study outcome was a binary variable proposed by IA2030, which defines children with 0-dose status as those aged 12 to 23 months who have not received the first dose of the DTP-containing vaccine (with 0 indicating receipt of the DTP1 and 1 indicating nonreceipt of the DTP1).^4^ Children aged 12 to 23 months who received the first dose of the DTP vaccine were considered vaccinated.^6^ The NFHS follows a standard procedure to collect vaccination data via trained field investigators. The information on the child’s vaccination status was transcribed from the vaccination card provided by the household. In cases in which the vaccination card was not available at the time of the interview, the child’s vaccination status was reported based on the primary caregiver’s recall.^10,18,19,20,21^

### Comparable States and UTs Across NFHS Rounds

Because the geographic boundaries of Indian states and districts evolved over the analytic period, we used information from the Integrated Public Use Microdata Series^23^ to reassign clusters within the corresponding district to the newly formed (or parent) state.^23^ We relied on administrative publications by the government of India to gather and track information on newly formed states (and the corresponding parent states) between 1993 and 2021 (details are available in eAppendix 2 in Supplement 1).^24,25^ Information on state-specific calibration and comparability across 5 rounds of the NFHS (NFHS-1 from 1992-1993, NFHS-2 from 1998-1999, NFHS-3 from 2005-2006, NFHS-4 from 2015-2016, and NFHS-5 from 2019-2021) is provided in eAppendix 2, eTable 1, and eTable 2 in Supplement 1. Information on the number of districts covered in each state for all survey rounds is provided in eTable 3 in Supplement 1.

### Statistical Analysis

We used data from all 5 NFHS rounds to examine national- and state-level estimates for children with 0-dose status. Given the differences in the sample size, the survey design, and the number and structure of geographic regions across NFHS rounds, repeated cross-sectional analyses were performed for descriptive and regression estimations. Because we used repeated cross-sectional analyses vs pooled analyses, descriptive data (eg, participant demographic characteristics expressed in numbers and percentages) for the 4 rounds were lengthy and therefore not reported in this article. Information on participant race and ethnicity was not reported because the NFHS does not collect these data.

For each of the 5 survey rounds, 36 states, and 8 UTs, we estimated the weighted prevalence of children with 0-dose status as the percentage of the total population of children. We also compared the distribution of children with 0-dose status across the states for all 5 rounds. Temporal patterns in state disparities were assessed via box plots and panel–data line plots. For these same states and UTs, we computed the annual absolute change (AAC) in the prevalence of children with 0-dose status (eAppendix 3 in Supplement 1). A negative AAC value represented a reduction in the prevalence, and a positive AAC value represented an increase in the prevalence. Because the baseline prevalence varied across states, we also computed relative change (in percentages) for all states (eAppendix 3 in Supplement 1). We used scatter plots and Pearson correlation coefficients to understand the patterns of association between 0-dose prevalence and AAC. We also generated prevalence and AAC maps to examine the geographic clustering of children with 0-dose status in India. We computed the state-level count of children with 0-dose status for the NFHS-5. To arrive at the target population, we calculated the state’s proportion of children aged 12 to 23 months in the total sample of children in the NFHS-5 and applied this proportion to 2021 state population projections of children aged 0 to 48 months.^26^ We then multiplied state-level weighted prevalence (NFHS-5) by the estimated population of children aged 12 to 23 months to arrive at the count.

For each of the 5 surveys, we used a 4-level logistic regression model to partition the total geographic variation in the prevalence of children with 0-dose status across children (level 1), cluster (level 2), district (level 3), and state (level 4) (eAppendix 4 in Supplement 1).^27^ Clusters are primary sampling units corresponding to villages in rural areas and blocks in urban areas. We then computed the variance partition coefficient (VPC) to assess the significance of each geographic unit (eAppendix 4 in Supplement 1). Multilevel regression analysis was not performed for the NFHS-3 because of the nonavailability of district identifiers in the sample design.

Multilevel modeling was performed using Stata software, version 15 (StataCorp LLC),^28^ and MLwiN software, version 3.0 (Centre for Multilevel Modelling, University of Bristol).^29,30^ The threshold for statistical significance was 2-tailed *P* = .05. All maps were generated using ArcGIS Pro, version 2.9.1 Esri).^31^ Appropriate shapefiles (geospatial vector data formats for geographic information systems software) for the 36 states and UTs were obtained from the International Institute for Population Sciences, the organization that administers the NFHS in India.

## Results

Records of 626 087 children younger than 5 years were examined for sample eligibility across the 5 NFHS rounds; 500 468 children were excluded (29 184 died, 470 590 were aged <12 months or >23 months, and 694 were missing information on vaccination status or geographic location). Descriptive and regression analyses included microdata for 125 619 live children aged 12 to 23 months (eFigure 1 in Supplement 1). The participation rate for all survey rounds was higher than 95.0%, typically ranging from 94.5% to 98.0% for individual and female respondents.^18,19,20,21^

**Figure 1.**
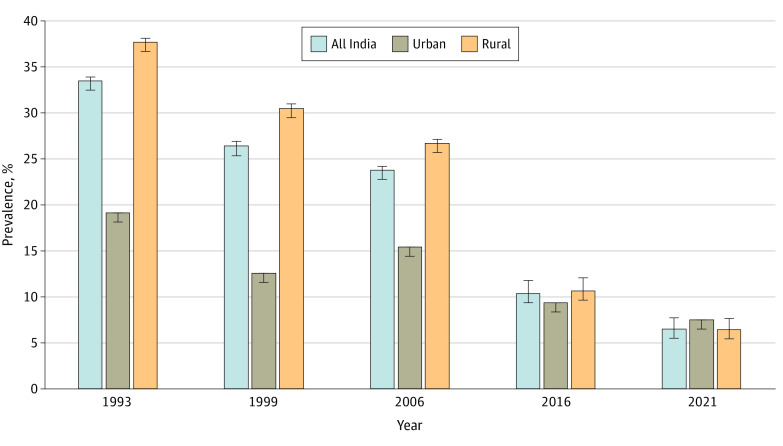
Prevalence of Children With 0-Dose Status in Rural and Urban India, 1993-2021 Among children aged 12 to 23 months based on data from 5 rounds of the National Family Health Survey (NFHS-1 from 1992-1993, NFHS-2 from 1998-1999, NFHS-3 from 2005-2006, NFHS-4 from 2015-2016, and NFHS-5 from 2019-2021).

**Figure 2.**
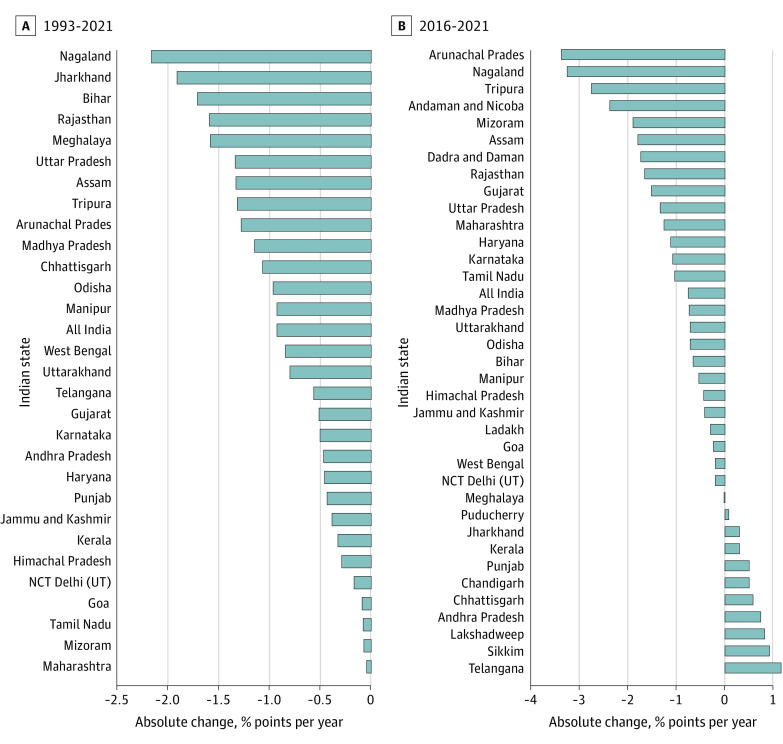
Annual Absolute Change in Prevalence of Children With 0-Dose Status by Indian State, 1993-2021 Based on data from 5 rounds of the National Family Health Survey (NFHS-1 from 1992-1993, NFHS-2 from 1998-1999, NFHS-3 from 2005-2006, NFHS-4 from 2015-2016, and NFHS-5 from 2019-2021). Negative and positive values represent decreases and increases in prevalence, respectively. NCT indicates national capital territory; and UT, union territory.

From 1993 to 2021, the national weighted prevalence of children with 0-dose status in India decreased by 26.8 percentage points, from 33.4% (95% CI, 32.5%-34.2%) in 1993 to 6.6% (95% CI, 6.4%-6.8%) in 2021 (Figure 1). The observed difference in the prevalence estimate between the 2 time points was statistically significant (*t* = 77.37; *df* = 58 188; *P* < .001). In terms of absolute burden, approximately 1.37 million children in 2021 were reported to have 0-dose vaccination status in India (eFigure 2 in Supplement 1). Disaggregated by area of residence, this number corresponds to a decrease from 37.6% to 6.3% in rural areas and from 19.2% to 7.5% in urban between 1993 and 2021 (eTable 4 in Supplement 1). The annual absolute decrease in 0-dose prevalence was 0.93 percentage points (Figure 2), and the annual relative decrease was 2.78% (eTable 5 in Supplement 1). The annual absolute decrease was highest at 1.33 percentage points for 2006 to 2016, followed by 1.01 percentage points for 1993 to 1999 and 0.76 percentage points for 2016 to 2021 (eTable 6 in Supplement 1). The annual relative decrease was highest (−7.38%) between 2016 and 2021. The absolute and relative reductions were higher for rural areas than urban areas for all survey rounds (eTable 7 and eTable 8 in Supplement 1).

An interactive view of the state maps and data is available via a dashboard.^32^ In 1993, the prevalence of children with 0-dose status was highest in Jharkhand (63.3%), Bihar (56.0%), Rajasthan (51.7%), Uttar Pradesh (48.5%), and all northeastern states (eg, Nagaland [78.8%], Meghalaya [62.9%], Arunachal Pradesh [49.7%], and Mizoram [16.4%]) (Table). Progress notwithstanding, in 2021, the prevalence of children with 0-dose status continued to be relatively high among the northeastern states of Meghalaya (17.0%), Nagaland (16.1%), Mizoram (14.3%), and Arunachal Pradesh (12.6%); prevalence in Assam was low (7.9%) (Table). Overall, 53.0% of children with 0-dose status resided in the populous states of Uttar Pradesh, Bihar, and Maharashtra in 2021. While all of the states experienced reductions in prevalence between 1993 and 2021, the annual absolute decrease was most rapid in the high-prevalence states of Nagaland (−2.16 percentage points), Jharkhand (−1.91 percentage points), Bihar (−1.71 percentage points), Rajasthan (−1.59 percentage points), and Uttar Pradesh (−1.34 percentage points) (Figure 2A). However, relative reductions in prevalence per year were highest in West Bengal (3.18%), followed by Rajasthan (−3.08%), Bihar (−3.05%), and Jharkhand (−3.02%) (eTable 5 in Supplement 1).

**Table.** Prevalence of Children With 0-Dose Status by State and Union Territory in India, 1993-2021^a^

Region	Children, No./total No. (%) [95% CI]				
	1993	1999	2006	2016	2021
India overall	3854/11 526 (33.4) [32.5 to 34.2]	2444/9292 (26.3) [25.4 to 27.1]	2237/9461 (23.6) [22.7 to 24.5]	5017/48 676 (10.3) [10.0 to 10.6]	3105/46 664 (6.6) [6.4 to 6.8]
**States**					
Andhra Pradesh	48/236 (20.3) [15.2 to 25.5]	20/193 (10.5) [6.1 to 14.9]	23/426 (5.5) [3.3 to 7.7]	17/567 (3.0) [1.6 to 4.4]	38/575 (6.7) [4.6 to 8.8]
Arunachal Pradesh	79/159 (49.7) [41.8 to 57.5]	50/118 (42.3) [33.2 to 51.3]	63/150 (42.1) [34.0 to 50.3]	245/847 (29.5) [26.4 to 32.7]	130/1032 (12.6) [10.5 to 14.8]
Assam	188/405 (46.5) [41.6 to 51.3]	125/309 (40.5) [35.0 to 46.0]	70/255 (27.6) [21.5 to 33.7]	323/1908 (16.9) [15.0 to 18.8]	167/2113 (7.9) [6.4 to 9.5]
Bihar	437/780 (56.0) [52.5 to 59.5]	399/661 (60.4) [56.7 to 64.2]	152/438 (34.8) [30.3 to 39.3]	471/4839 (9.7) [8.9 to 10.6]	280/4308 (6.4) [5.6 to 7.1]
Chhattisgarh	55/154 (35.8) [28.2 to 43.5]	34/101 (33.7) [24.3 to 43.1]	32/273 (11.8) [7.9 to 15.6]	29/1529 (1.9) [1.2 to 2.6]	83/1740 (4.8) [3.7 to 5.8]
Goa	17/278 (6.1) [3.3 to 8.9]	2/121 (1.7) [−0.6 to 4.1]	6/204 (3.2) [0.5 to 5.9]	4/87 (4.7) [−0.2 to 9.7]	3/75 (3.5) [−2.3 to 9.2]
Gujarat	103/470 (21.9) [18.2 to 25.7]	59/365 (16.2) [12.4 to 20.0]	57/305 (18.7) [14.3 to 23.0]	203/1391 (14.6) [12.7 to 16.5]	143/2035 (7.0) [5.8 to 8.2]
Haryana	87/451 (19.3) [15.6 to 22.9]	34/325 (10.5) [7.1 to 13.8]	36/227 (15.9) [11.1 to 20.7]	173/1495 (11.6) [9.9 to 13.2]	85/1411 (6.0) [4.7 to 7.3]
Himachal Pradesh	33/338 (9.9) [6.7 to 13.1]	9/282 (3.3) [1.2 to 5.5]	6/189 (3.4) [0.8 to 6.0]	21/561 (3.7) [2.1 to 5.2]	10/574 (1.5) [0.5 to 2.6]
Jharkhand	51/80 (63.3) [52.5 to 74.1]	91/166 (55.0) [47.3 to 62.6]	100/295 (33.8) [28.4 to 39.2]	152/2377 (6.4) [5.4 to 7.4]	165/2079 (7.9) [6.7 to 9.2]
Karnataka	105/545 (19.3) [15.9 to 22.6]	49/403 (12.1) [8.9 to 15.3]	54/406 (13.3) [10.0 to 16.7]	147/1467 (10.1) [8.5 to 11.7]	80/1709 (4.7) [3.7 to 5.8]
Kerala	58/393 (14.8) [11.2 to 18.3]	10/231 (4.5) [1.8 to 7.2]	8/215 (3.9) [1.0 to 6.7]	18/486 (3.8) [2.1 to 5.6]	29/544 (5.3) [3.3 to 7.4]
Madhya Pradesh	287/725 (39.5) [36.0 to 43.1]	271/709 (38.2) [34.6 to 41.8]	122/520 (23.5) [19.8 to 27.1]	447/4519 (9.9) [9.0 to 10.8]	205/3299 (6.2) [5.3 to 7.0]
Maharashtra	46/494 (9.3) [6.7 to 11.9]	22/540 (4.1) [2.4 to 5.8]	36/604 (6.0) [4.1 to 7.9]	249/1750 (14.3) [12.6 to 15.9]	158/1974 (8.0) [6.7 to 9.3]
Manipur	43/127 (33.9) [25.5 to 42.2]	47/200 (23.5) [17.6 to 29.4]	83/334 (25.0) [20.1 to 29.9]	111/1142 (9.7) [7.9 to 11.4]	44/627 (7.0) [4.9 to 9.2]
Meghalaya	90/143 (62.9) [54.9 to 70.9]	90/163 (55.4) [47.7 to 63.1]	75/204 (36.9) [30.2 to 43.6]	143/835 (17.1) [14.5 to 19.7]	211/1233 (17.0) [14.8 to 19.3]
Mizoram	18/110 (16.4) [9.3 to 23.4]	18/158 (11.6) [6.6 to 16.7]	15/151 (10.3) [5.4 to 15.2]	235/989 (23.8) [21.1 to 26.4]	73/512 (14.3) [11.1 to 17.4]
Nagaland	126/160 (78.8) [72.3 to 85.2]	72/133 (54.1) [45.5 to 62.7]	219/418 (52.3) [47.5 to 57.1]	286/884 (32.4) [29.3 to 35.6]	98/612 (16.1) [13.1 to 19.2]
Odisha	156/505 (30.9) [26.9 to 34.9]	83/446 (18.6) [15.0 to 22.3]	53/332 (16.0) [12.0 to 20.0]	136/2089 (6.5) [5.4 to 7.5]	49/1681 (2.9) [2.1 to 3.8]
Punjab	63/349 (18.1) [14.0 to 22.1]	30/252 (11.8) [7.8 to 15.8]	30/234 (12.7) [8.4 to 17.0]	30/1017 (3.0) [2.0 to 4.1]	61/1113 (5.5) [4.1 to 6.9]
Rajasthan	334/646 (51.7) [47.8 to 55.6]	380/770 (49.3) [45.8 to 52.9]	126/361 (34.9) [30.0 to 39.9]	430/3128 (13.8) [12.6 to 15.0]	148/2697 (5.5) [4.6 to 6.4]
Sikkim	NA	32/138 (23.0) [15.9 to 30.1]	7/131 (5.1) [1.3 to 8.9]	1/206 (0.6) [−0.5 to 1.6]	6/126 (5.2) [1.0 to 9.5]
Tamil Nadu	20/420 (4.8) [2.7 to 6.8]	5/427 (1.1) [0.1 to 2.1]	3/308 (1.1) [−0.1 to 2.3]	119/1550 (7.7) [6.3 to 9.0]	35/1390 (2.5) [1.7 to 3.4]
Telangana	44/182 (24.2) [17.9 to 30.5]	22/186 (11.8) [7.2 to 16.5]	NA	9/470 (2.0) [0.7 to 3.2]	124/1578 (7.8) [6.4 to 9.2]
Tripura	52/121 (43.0) [34.0 to 51.9]	27/93 (28.9) [19.5 to 38.3]	23/117 (19.6) [12.2 to 27.0]	46/245 (18.6) [13.2 to 24.0]	20/422 (4.8) [2.4 to 7.1]
Uttar Pradesh	812/1673 (48.5) [46.1 to 50.9]	323/805 (40.1) [36.7 to 43.5]	548/1252 (43.8) [41.0 to 46.6]	1256/7674 (16.4) [15.5 to 17.2]	681/7004 (9.7) [9.0 to 10.5]
Uttarakhand	68/245 (27.9) [22.2 to 33.5]	8/67 (11.7) [3.8 to 19.6]	37/215 (17.1) [12.1 to 22.2]	904/1089 (8.3) [6.6 to 9.9]	36/754 (4.7) [3.2 to 6.3]
West Bengal	136/511 (26.6) [22.7 to 30.4]	86/384 (22.4) [18.2 to 26.6]	39/435 (9.0) [6.1 to 11.9]	34/1102 (3.1) [2.0 to 4.2]	24/1165 (2.1) [1.1 to 3.1]
**Union territories**					
National capital territory of Delhi	47/459 (10.2) [7.5 to 13.0]	19/246 (7.8) [4.4 to 11.1]	35/216 (16.1) [11.2 to 21.1]	19/301 (6.3) [3.6 to 9.1]	33/618 (5.3) [3.4 to 7.2]
Jammu and Kashmir	60/367 (16.3) [12.5 to 20.1]	37/300 (12.2) [8.5 to 16.0]	35/246 (14.1) [8.2 to 19.9]	103/1449 (7.2) [5.4 to 8.9]	57/1075 (5.1) [3.6 to 6.7]
Andaman and Nicobar	NA	NA	NA	18/131 (14.1) [7.9 to 20.2]	2/100 (2.2) [−1.1 to 5.5]
Chandigarh	NA	NA	NA	2/37 (4.1) [−2.6 to 10.9]	2/29 (6.6) [−3.2 to 16.4]
Dadra and Nagar Haveli; Daman and Diu	NA	NA	NA	15/140 (10.9) [5.6 to 16.2]	3/161 (2.2) [−0.2 to 4.6]
Lakshadweep	NA	NA	NA	2/59 (3.2) [−1.4 to 7.8]	3/45 (7.3) [−0.9 to 15.6]
Ladakh	NA	NA	NA	2/125 (1.5) [−0.8 to 3.7]	0/89 (0) [0 to 0]
Puducherry	NA	NA	NA	1/191 (0.5) [−0.5 to 1.4]	1/165 (0.9) [−0.6 to 2.5]

Abbreviation: NA, not available.

^a^ Among children aged 12 to 23 months based on data from 5 rounds of the National Family Health Survey (NFHS-1 from 1992-1993, NFHS-2 from 1998-1999, NFHS-3 from 2005-2006, NFHS-4 from 2015-2016, and NFHS-5 from 2019-2021).

A total of 10 states experienced an increase in 0-dose prevalence between 2016 and 2021 (Figure 2B). For example, the AAC between 2016 and 2021 increased in Telangana (1.16 percentage points), Sikkim (0.92 percentage points), and Andhra Pradesh (0.74 percentage points), reflecting an increase in prevalence (Figure 2B). For most states, the relative reduction in prevalence was higher in rural areas than in urban areas (eTable 8 in Supplement 1). The spatial clustering of 0-dose prevalence across all Indian states is shown in eFigure 3 in Supplement 1. Overall, between 1993 and 2021, the relative reductions were comparatively higher in the central regions of India (eFigure 4 in Supplement 1). The estimated count of children with 0-dose status was highest in populous states like Uttar Pradesh (420 022 children) and Bihar (160 000 children) (the estimated count of children with 0-dose status across states is shown in eFigure 2 in Supplement 1). These 2 states together comprised one-third of the sample of children with 0-dose status in India in 2021 (the distribution of the sample of children with 0-dose status across states is shown in eTable 9 in Supplement 1). Estimated counts of children with 0-dose status were also high in Maharashtra (125 603 children), Madhya Pradesh (99 818 children), Gujarat (75 952 children), Rajasthan (72 750 children), and Jharkhand (55 809 children). Together, these 7 states accounted for 64.6% of children with 0-dose status in India in 2021.

**Figure 3.**
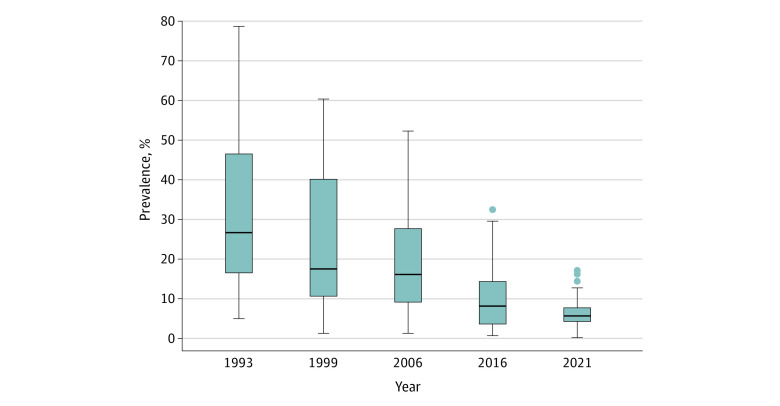
Prevalence of Children With 0-Dose Status Across Indian States, 1993-2021 Based on data from 5 rounds of the National Family Health Survey (NFHS-1 from 1992-1993, NFHS-2 from 1998-1999, NFHS-3 from 2005-2006, NFHS-4 from 2015-2016, and NFHS-5 from 2019-2021). Box and whisker plot showing the variability of a data set using lowest and highest values as well as quartiles of data. Lower and upper whiskers represent minimum and maximum values, respectively. Lines at the lower and upper ends of the boxes represent 25th percentiles and 75th percentiles, respectively. The line within each box represents the median (ie, quartile 2 or 50th percentile).

Relative disparities across states decreased substantially over the 5 survey rounds toward converging prevalence levels (Figure 3). The median (IQR) of 0-dose prevalence across states decreased from 27.3% (16.4%-46.5%) in 1993 to 5.8% (4.7%-7.8%) in 2021. This shift toward convergence across states was also confirmed in panel–data line plots and was visible in both rural and urban areas (eFigure 5 in Supplement 1). We found a high correlation (*r* = −0.98) between the baseline prevalence and absolute reductions across the states between the 2 time points (1993 and 2021) (eFigure 6 in Supplement 1). Scatter plots revealed a null to moderate correlation for 1993 to 1999 (*r* = 0.27) and 1999 to 2006 (*r* = 0.68), whereas a higher correlation was observed for more recent survey rounds (2006-2016: *r* = 0.82; 2016-2021: *r* = 0.87) (eFigure 7 in Supplement 1).

**Figure 4.**
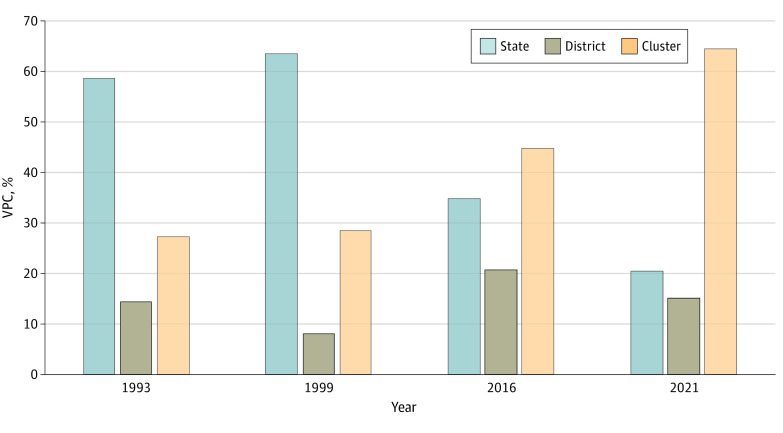
Partitioning Variation Among Children With 0-Dose Status in Multiple Geographic Regions in India, 1993-2021 Based on data from 5 rounds of the National Family Health Survey (NFHS-1 from 1992-1993, NFHS-2 from 1998-1999, NFHS-3 from 2005-2006, NFHS-4 from 2015-2016, and NFHS-5 from 2019-2021). Variance partition coefficients (VPCs) for 2006 from the NFHS-3 were not estimated due to the nonavailability of district identifiers in the data.

In the initial survey rounds, states were the highest source of geographic variation, with a share of 58.4% in 1993 (VPC [SE], 1.71 [0.60]) and 63.3% in 1999 (VPC [SE], 2.47 [0.81]) (Figure 4; eTable 10 in Supplement 1). However, clusters accounted for the highest share in more recent surveys from 2016 (44.7%; VPC [SE], 1.04 [0.32]) and 2021 (64.3%; VPC [SE], 0.38 [0.12]). States accounted for approximately 34.7% (VPC [SE], 1.34 [0.07]) and 20.5% (VPC [SE], 1.19 [0.07]) of the total geographic variation in children with 0-dose status in 2016 and 2021, respectively. For all 5 survey rounds, the share of interdistrict variation was consistently the lowest of all of the analyzed geographic regions (eTable 10 in Supplement 1). These patterns were consistent when considering 95% coverage boundaries for probability across each geographic region (eTable 11 in Supplement 1).

## Discussion

To our knowledge, this repeated cross-sectional study is the first investigation of children with 0-dose status to examine temporal patterns and inequalities in vaccination across all states in India using 5 cross-sectional surveys spanning 29 years (1993-2021). We had 6 salient findings. First, over the last 3 decades, the national prevalence of children with 0-dose status decreased in absolute terms by 26.8 percentage points, reaching 6.6% (equivalent to approximately 1.37 million children with 0-dose status) in 2021. Due to greater prevalence reductions in rural areas, the historical urban advantage was eliminated. Second, all states experienced a reduction in prevalence from 1993 to 2021. Substantial reductions in interstate inequalities were observed over time, with all of the states and UTs converging toward substantially lower prevalence levels.

Third, despite substantial progress, interstate inequalities remained. In 2021, the highest prevalence of unvaccinated children was in the northeastern states of Meghalaya, Nagaland, Mizoram, and Arunachal Pradesh, while the highest counts of unvaccinated children were in Uttar Pradesh, Bihar, Maharashtra, Madhya Pradesh, Gujarat, Rajasthan, and Jharkhand, together accounting for 64.6% of children with 0-dose status in India in 2021. Fourth, findings suggested that relative decreases were most important during the 2 most recent survey rounds, which correlated with policy periods during which important policy actions were undertaken by the government of India. Fifth, 10 states experienced a resurgence in children with 0-dose status between the 2 most recent surveys (2015-2016 and 2019-2021), including several traditionally high-performing states and UTs, such as Punjab, Sikkim, Kerala, Puducherry, and Chandigarh. Sixth, multilevel analysis found that the variance share of states decreased over time, while intercluster variability increased substantially, with clusters representing the largest share of total variation across geographic regions in 2021.

India’s notable progress in reaching children with 0-dose status corroborates the importance of major national government initiatives. The absolute decrease in 0-dose prevalence was greatest between the NFHS-3 (2005-2006) and the NFHS-4 (2015-2016) surveys, when the National Rural Health Mission was established, while the relative reduction was highest between the 2 most recent surveys (NFHS-4 [2015-2016] and NFHS-5 [2019-2021]), during which major national initiatives to improve immunization coverage (the MI and IMI programs) were introduced.^17^ A high correlation between the baseline prevalence and absolute change reflected greater reductions in high-prevalence states between the 2 most recent surveys, suggesting that initiatives of the government of India have been well targeted.^17^ Furthermore, the observed decrease in the absolute number of children with 0-dose status is a testament to the successful targeting strategy (ie, including the counts of children with 0-dose status for resource allocation) implemented under the IMI program.^17^ The elimination of the rural-urban gap further reflected enhanced delivery systems in remote parts of the country.

Of note, some states and UTs experienced an increase in the prevalence of children with 0-dose status between 2016 and 2021. One of the plausible explanations is the negative implications of pandemic-induced disturbances for routine vaccination.^1^ Notably, the more recent increase in prevalence was observed in states such as Kerala, Punjab, Andhra Pradesh, and Telangana. This finding pointed precisely to the salience of promoting sustainable efforts that can maintain vaccination coverage over time, even in states with a lower burden of children with 0-dose status. To address lapses in immunization due to the COVID-19 pandemic, in February 2022, the government of India announced the IMI 4.0 program, to be conducted in 416 districts and 33 states and UTs across the country.

We observed that the burden among children from northeastern states (with the exception of Assam) remained relatively high. These states also had low levels of full immunization coverage, antenatal (and postnatal) maternal care, and a high incidence of vaccine-preventable diseases.^10,33,34^ Notably, these states have distinct geographic placement in India, with connectivity to the rest of the country by only a narrow corridor of land. Furthermore, in addition to administrative bottlenecks, these states are also characterized by intertribal conflicts, higher interstate immigration, and cultural barriers. Some of the possible pathways to expand vaccination coverage may be associated with promoting greater awareness among female respondents, particularly those representing socially vulnerable groups like scheduled castes and scheduled tribes (officially designated groups of people who are among the most socioeconomically disadvantaged in India). In addition to improving educational levels, interventions supporting women’s empowerment and the enhancement of active health-seeking behavior can also be potential solutions.^33,34^

In absolute terms, the states of Uttar Pradesh, Bihar, Madhya Pradesh, Rajasthan, Maharashtra, and Gujarat comprise the majority of children with 0-dose status in India. Notably, in states like Bihar and bordering areas, natural calamities (mainly floods) severely affect the health care delivery system, with 22 of 38 districts in the state vulnerable to frequent flooding.^35^ Because climate change threatens to aggravate extreme weather events, it will be important to address and include flood mitigation strategies in the policy framework to enhance vaccination coverage in these high-burden states. The findings from the multilevel analysis suggested a required shift in focus to targeting smaller geographic units (villages and blocks) with high 0-dose burden. Data from the most recent survey (2019-2021) suggested that intercluster variation was highest across all geographic regions and warrants greater policy attention. The MI and IMI programs have undertaken increasingly fine-tuned targeting approaches for these smaller geographic regions, which will be important to ensure that high-burden villages (blocks) within low-prevalence districts or states are not overlooked.

### Limitations

This study has several limitations. First, the survey design of the NFHS-5 (2019-2021) was broadly based on the Census 2011 sampling framework, which may be outdated. In addition, the household survey design may not capture all children with 0-dose status. Unstable housing areas like slums are less likely to be covered in the survey. Furthermore, the exclusion of dead children in assessing immunization coverage may lead to survivorship bias, thereby producing an underestimation of the true burden. Although the geographic coverage of the NFHS has improved substantially over time, data for most UTs were available only for the NFHS-4 (2015-2016) and the NFHS-5 (2019-2021).

Second, due to the lack of availability of district identifiers in the NFHS-3 (2005-2006), prevalence estimation for newly formed states was not possible for this survey round. For the same reason, we did not include the NFHS-3 in multilevel analyses; however, this exclusion does not alter the interpretation of the overall change between 1993 and 2021. Third, a notable part of the data on child immunization was reported via caregiver recall, which may result in misclassification. Fourth, the IA2030 definition is a proxy for lack of access to routine immunization, but studies comparing alternative definitions have suggested that the IA2030 definition is robust.^11^

Fifth, due to a lack of more recent data, our count estimates are based on state population projections by the Census of India from 2011. Sixth, the Covid-19 pandemic has substantially changed the vaccination picture worldwide and in India, with an estimated decrease in DTP1 prevalence from 94% to 88% and an associated increase in the number of children with 0-dose status in India to more than 3 million.^9^ The NFHS-5 data collection was interrupted by the COVID-19 pandemic, with some states surveyed in 2019 and others in 2021. Due to the timing of NFHS-5 data collection by state and the fact that immunization data for infants are collected retrospectively for the age 12- to 23-month window, the implications of the pandemic for routine immunization among infants aged 0 to 12 months are not fully captured in the NFHS-5 survey data.

## Conclusions

The findings of this repeated cross-sectional study support the need for sustained efforts to target the northeastern parts of India and high-burdened states like Uttar Pradesh and Bihar. Immunization is the first line of defense against childhood mortality; therefore, reducing the prevalence of children with 0-dose status is an important first step. The observed resurgence of 0-dose prevalence in low-prevalence states highlights the importance of ongoing programs like IMI 4.0. Prioritizing small administrative units such as villages will be important to further India’s efforts to ensure that every child has timely access to these life-saving and life-shaping vaccines.

## Notes

Supplement 1. **eAppendix 1.** National Family Health Survey: Summary of All Survey Rounds **eFigure 1.** Construction of the Analytic Sample **eAppendix 2.** Construction of Comparable States and Union Territories Across NFHS Rounds **eTable 1.** Overview of Data Comparability for NFHS Round by State or Union Territory in India **eTable 2.** Summary of the Method Used for Creating State Equivalence for Each NFHS Survey **eTable 3.** Number of Districts From NFHS-1 and NFHS-2 That Fall Into the Current States **eAppendix 3.** Statistical Analysis: Annual Absolute and Relative Change **eAppendix 4.** Statistical Analysis: Variance Partitioning: 4-Level Regression Model **eFigure 2.** Estimated Count of 0-Dose Children (Aged 12-23 Months) by States in India, NFHS 2021 **eTable 4.** Prevalence of 0-Dose Children (Aged 12-23 Months) by States and Union Territories and Rural and Urban Areas in India, NFHS 1993-2021 **eTable 5.** Annual Relative Change (Percentage) in 0-Dose Children Across States and Union Territories in India, NFHS 1993-2021 **eTable 6.** Annual Absolute Change (Percentage Points) in the Prevalence of 0-Dose Children by States in India, NFHS 1993-2021 **eTable 7.** Annual Absolute Change (Percentage Points) in 0-Dose Children Across States and Union Territories and Rural and Urban Areas in India, NFHS 1993-2021 **eTable 8.** Annual Relative Change (Percentage) in 0-Dose Children Across States and Union Territories and Rural and Urban Areas in India, NFHS 1993-2021 **eTable 9.** Distribution (Percentage) of 0-Dose Children Across States and Union Territories in India, NFHS 1993-2021 **eFigure 3.** Maps for the Prevalence of 0-Dose Children (Aged 12-23 Months) by State in India, NFHS 1993-2021 **eFigure 4.** Maps for Annual Absolute Change (Percentage Points per Year) in the Prevalence of 0-Dose Children Across States and Union Territories in India, NFHS 1993-2021 **eFigure 5.** Panel-Line Plots for the Prevalence of 0-Dose Children (Aged 12-23 Months) Across States, Rural and Urban Areas, and All India, NFHS 1993-2021 **eFigure 6.** Scatter Plot for Correlation Between Baseline Prevalence and Annual Absolute Change (Percentage Points) in the Prevalence of 0-Dose Children in India, NFHS 1993-2021 **eFigure 7.** Scatter Plot for Correlation Between Baseline Prevalence and Annual Absolute Change (Percentage Points) in the Prevalence of 0-Dose Children in India, NFHS 1992-2021 **eTable 10.** Variance Partition Coefficients (VPCs) (Percentage) in 0-Dose Children by Multiple Geographic Regions in India, NFHS 1993-2021 **eTable 11.** Mean Probability of 0-Dose Children With 95% Coverage Boundaries Across Geographic Regions in India, NFHS 1993-2021

Supplement 2.
Data Sharing Statement
