# Supplementary material for: Patterns in the Prevalence of Unvaccinated Children Across 36 States and Union Territories in India, 1993-2021
Source: JAMA Netw Open. 2023 Feb 10;6(2):e2254919. doi: 10.1001/jamanetworkopen.2022.54919 (PMC9918883; doi:10.1001/jamanetworkopen.2022.54919)
Supplement: Supplement 1. — eAppendix 1. National Family Health Survey: Summary of All Survey Rounds eFigure 1. Construction of the Analytic Sample eAppendix 2. Construction of Comparable States and Union Territories Across NFHS Rounds eTable 1. Overview of Data Comparability for NFHS Round by State or Union Territory in India eTable 2. Summary of the Method Used for Creating State Equivalence for Each NFHS Survey eTable 3. Number of Districts From NFHS-1 and NFHS-2 That Fall Into the Current States eAppendix 3. Statistical Analysis: Annual Absolute and Relative Change eAppendix 4. Statistical Analysis: Variance Partitioning: 4-Level Regression Model eFigure 2. Estimated Count of 0-Dose Children (Aged 12-23 Months) by States in India, NFHS 2021 eTable 4. Prevalence of 0-Dose Children (Aged 12-23 Months) by States and Union Territories and Rural and Urban Areas in India, NFHS 1993-2021 eTable 5. Annual Relative Change (Percentage) in 0-Dose Children Across States and Union Territories in India, NFHS 1993-2021 eTable 6. Annual Absolute Change (Percentage Points) in the Prevalence of 0-Dose Children by States in India, NFHS 1993-2021 eTable 7. Annual Absolute Change (Percentage Points) in 0-Dose Children Across States and Union Territories and Rural and Urban Areas in India, NFHS 1993-2021 eTable 8. Annual Relative Change (Percentage) in 0-Dose Children Across States and Union Territories and Rural and Urban Areas in India, NFHS 1993-2021 eTable 9. Distribution (Percentage) of 0-Dose Children Across States and Union Territories in India, NFHS 1993-2021 eFigure 3. Maps for the Prevalence of 0-Dose Children (Aged 12-23 Months) by State in India, NFHS 1993-2021 eFigure 4. Maps for Annual Absolute Change (Percentage Points per Year) in the Prevalence of 0-Dose Children Across States and Union Territories in India, NFHS 1993-2021 eFigure 5. Panel-Line Plots for the Prevalence of 0-Dose Children (Aged 12-23 Months) Across States, Rural and Urban Areas, and All India, NFHS 1993-2021 eFigure 6. Scatter P [file jamanetwopen-e2254919-s001.pdf]

Supplemental Online Content

Rajpal S, Kumar A, Johri M, Kim R, Subramanian SV. Patterns in the prevalence of unvaccinated children across 36 states and union territories in India, 1993-2021. *JAMA Netw Open*. 2023;6(2):e2254919. doi:10.1001/jamanetworkopen.2022.54919

eAppendix 1. National Family Health Survey: Summary of All Survey Rounds

eFigure 1. Construction of the Analytic Sample

eAppendix 2. Construction of Comparable States and Union Territories Across NFHS Rounds

eTable 1. Overview of Data Comparability for NFHS Round by State or Union Territory in India

eTable 2. Summary of the Method Used for Creating State Equivalence for Each NFHS Survey

eTable 3. Number of Districts From NFHS-1 and NFHS-2 That Fall Into the Current States

eAppendix 3. Statistical Analysis: Annual Absolute and Relative Change

eAppendix 4. Statistical Analysis: Variance Partitioning: 4-Level Regression Model

eFigure 2. Estimated Count of 0-Dose Children (Aged 12-23 Months) by States in India, NFHS 2021

eTable 4. Prevalence of 0-Dose Children (Aged 12-23 Months) by States and Union Territories and Rural and Urban Areas in India, NFHS 1993-2021

eTable 5. Annual Relative Change (Percentage) in 0-Dose Children Across States and Union Territories in India, NFHS 1993-2021

eTable 6. Annual Absolute Change (Percentage Points) in the Prevalence of 0-Dose Children by States in India, NFHS 1993-2021

eTable 7. Annual Absolute Change (Percentage Points) in 0-Dose Children Across States and Union Territories and Rural and Urban Areas in India, NFHS 1993-2021

eTable 8. Annual Relative Change (Percentage) in 0-Dose Children Across States and Union Territories and Rural and Urban Areas in India, NFHS 1993-2021

eTable 9. Distribution (Percentage) of 0-Dose Children Across States and Union Territories in India, NFHS 1993-2021

eFigure 3. Maps for the Prevalence of 0-Dose Children (Aged 12-23 Months) by State in India, NFHS 1993-2021

eFigure 4. Maps for Annual Absolute Change (Percentage Points per Year) in the Prevalence of 0-Dose Children Across States and Union Territories in India, NFHS 1993-2021

eFigure 5. Panel-Line Plots for the Prevalence of 0-Dose Children (Aged 12-23 Months) Across States, Rural and Urban Areas, and All India, NFHS 1993-2021

eFigure 6. Scatter Plot for Correlation Between Baseline Prevalence and Annual Absolute Change (Percentage Points) in the Prevalence of 0-Dose Children in India, NFHS 1993-2021

© 2023 Rajpal S et al. *JAMA Network Open*.

eFigure 7. Scatter Plot for Correlation Between Baseline Prevalence and Annual Absolute Change (Percentage Points) in the Prevalence of 0-Dose Children in India, NFHS 1992-2021

eTable 10. Variance Partition Coefficients (VPCs) (Percentage) in 0-Dose Children by Multiple Geographic Regions in India, NFHS 1993-2021

eTable 11. Mean Probability of 0-Dose Children With 95% Coverage Boundaries Across Geographic Regions in India, NFHS 1993-2021

This supplemental material has been provided by the authors to give readers additional information about their work.

© 2023 Rajpal S et al. *JAMA Network Open*.

eAppendix 1. National Family Health Survey: Summary of All Survey Rounds

The NFHS is part of the Demographic and Health Surveys (DHS) Program which was initiated in the 1992-93 to provide estimates of important indicators on population, health and nutrition for India, and has conducted more than 400 surveys in over 90 countries. The NFHS surveys are conducted with the aim to provide essential data on health and family welfare and on key emerging issues. Over time, the NFHS has expanded its scope of indicator coverage pertaining to several development themes, including gender-based issues such as women's autonomy and experience of domestic violence, health services quality, sexually transmitted infections, and HIV/AIDS. Further, recent surveys also cover information related to marital and sexual relationships and living arrangements, non-communicable diseases and behavioral risk factors.

All five waves of the NFHS surveys covered geographic areas comprising more than 99% of India's population. The sampling approach was refined over survey rounds to achieve increasingly fine geographic representation. In the NFHS-1 (1992-93), the goal was to generate national and state-level estimates, in the NFHS-2 (1998-99), to generate national and state-level estimates, rural-urban estimates for most states, regional estimates for four states, and estimates for three major cities, and in the NFHS-3 (2005-06), to generate national and state-level estimates, rural-urban estimates for all states, and estimates for the slum and non-slum populations of eight major cities. The NFHS-4 (2015-16) was designed to provide estimates for all 29 states, 7 union territories, and 640 districts, as well as the slum populations of 8 major cities. Of note, while the NFHS-1 included ever-married women aged 13-49 and the NFHS-2 ever-married women aged 15-49, the NFHS-3 and NFHS-4 included women aged 15-49 irrespective of marital status. District level representation was first achieved in the NFHS-4, which derived a representative sample for all 640 districts within India's 29 states and 7 union territories.

Although geographic coverage is excellent, some areas, including conflict-affected, remote, or small, low-density settings, were excluded from the surveys. The NFHS-1 was conducted uniformly in all states except Kashmir, Sikkim and some small union territories. The NFHS-2 did not include Tripura or small union territories. The NFHS-3 included all states, and the NFHS-4 included all states and union territories. The NFHS surveys divide populations by urban and rural area of residence and use the most recent available national census data to define the sampling frame. In rural areas, for all survey rounds, a representative sample of households was constructed through stratified, probabilistic two-stage sampling. In the first stage, Primary Sampling Units (PSUs) corresponding to villages were stratified on key variables of interest and selected by probability proportional to PSU size.

© 2023 Rajpal S et al. *JAMA Network Open.*

Enumeration (household listing) was performed in selected PSUs (or, in the case of large PSUs, in PSU segments). In the second stage, within PSUs, households were selected from the household lists using systematic sampling with equal probability. Urban areas followed a similar procedure with two salient differences: urban PSUs correspond to census enumeration blocks, and a mix of two-stage (NFHS-4 & NFHS-5), three-stage (NFHS-I, NFHS- 2), and two- and three-stage (NFHS-3) sampling approaches were employed.

The sample households for the surveys were (number of households) NFHS-1 (88,562), NFHS-2 (92,486), NFHS-3 (109,041), NFHS-4 (609,120) and NFHS-5 (636,699). The NFHS-4 and NFHS-5 surveys size was substantially larger than the other three rounds (individually and combined). The DHS Programme states: "The figures of NFHS-4 and that of earlier rounds may not be strictly comparable due to differences in sample size and NFHS-4 will be a benchmark for future surveys." https://dhsprogram.com/methodology/survey/survey-display-355.cfm It should be noted that the NFHS-4 survey used districts as defined in Census 2011 and does not represent the new districts created after that date. For this reason, the NFHS-4 and NFHS-5 are the only surveys for which it is possible to produce comparable state level estimates directly. The NFHS surveys used a multistage design with stratified sampling for specific groups of interest, including scheduled castes and tribes, and women with low levels of literacy. We used the standard DHS sample weights procedure to ensure that prevalence estimates were representative of the population. As is standard in vaccination coverage surveys, in the NFHS series, vaccination data is collected from two sources, the vaccination card and caregiver recall. For NFHS-1, identifier information for 389 out of 392 surveyed districts (within 25 states) was provided by IPUMS. For NFHS-2, IPUMS provided information on all 440 surveyed districts. For NFHS-3, however, no information on district identifiers was provided, thereby restricting state comparability for that round. Both NFHS-4 and NFHS-5 have district-representative samples nested within the 36 comparable states and UTs.

© 2023 Rajpal S et al. *JAMA Network Open.*

eFigure 1. Construction of the Analytic Sample

Flow diagram showing exclusions and final sample sizes for primary analysis of the study population, Indian National Family Health Survey 1993, 1999, 2006, 2016, & 2021

Original Sample Size

NFHS1	NFHS2	NFHS3	NFHS4	NFHS5
48959	33026	51555	259,627	232920

Exclusion – Dead Children

NFHS1	NFHS2	NFHS3	NFHS4	NFHS5
3680	2042	2876	11884	8702

Alive Children

NFHS1	NFHS2	NFHS3	NFHS4	NFHS5
45279	30984	48679	236455	224218

Exclusion – Children below 12 months & above 23 months

Rounds	Below 12 months	Above 23 months
NFHS1	11920	21759
NFHS2	9789	11717
NFHS3	9561	29592
NFHS4	47854	150961
NFHS5	40732	136705

Eligible Children – aged 12-23 months

NFHS1	NFHS2	NFHS3	NFHS4	NFHS5
11600	9478	9526	48928	46781

Exclusion – Missing/Flagged/Geography Unmatched Cases

Criteria	NFHS1	NFHS2	NFHS3	NFHS4	NFHS5
Unmatched District Geography	26	73		-	-
DPT1	48	113	65	252	117

Final Analytic Sample Children (12-23 Months)

NFHS1	NFHS2	NFHS3	NFHS4	NFHS5
11526	9292	9461	48676	46664

© 2023 Rajpal S et al. *JAMA Network Open*.

eAppendix 2. Construction of Comparable States and Union Territories Across NFHS Rounds

This method relies on assigning all clusters within a particular district to a new state that was formed from a parent state that was surveyed in earlier NFHS surveys. The NFHS-1 surveyed 392 districts in 25 states (at the time of the survey). Out of the 392 districts, 389 of them are identified by IPUMS. The NFHS-2 surveyed 440 districts in 26 states (at time of the survey) and out of the 440 districts, all of them were identified by IPUMS.

This identification was possible because Demographic and Health Surveys (DHS) released district codes in the NFHS-1 and NFHS-2 that matched the district's sampling frame codes. In the NFHS-3, DHS implemented a scrambling process that prevented the identification of districts. Both the NFHS-4 and NFHS-5 were district representative surveys and therefore had publicly available district codes. The NFHS-1 used the 1981 census sampling frame for rural areas, except for Assam, Delhi, and Punjab, which used the 1991 census as their sampling frame. In urban areas, the 1991 census was used except for Andhra Pradesh, Himachal Pradesh, Madhya Pradesh, Tamil Nadu, and West Bengal, which used the National Sample Survey Organization's (NSSO) sampling frame. The NFHS-2 used the 1991 census sampling frame for rural and urban areas. For the NFHS-1 and NFHS-2 we used the Registrar General and Census Commissioner of India's "India Administrative Atlas 1872 – 2001: A Historical Perspective of Evolution of Districts and States" to track the formation of new states and districts. Additionally, this method uses the Andhra Pradesh Reorganization Act of 2014 and the Jammu and Kashmir Reorganization Act of 2019 to identify the districts that fell into Telangana and Ladakh, respectively. To confirm that no districts were reassigned to a different state between 2001 and 2011, we used the Census of India's Administrative Atlas of 2011 to identify how the 593 districts of Census 2001 linked to the 640 districts in Census 2011.

The NFHS surveys were not designed with the intention that the states would be reconfigured in the future, and because of this, the results generated from this method may not be fully state representative. We have outlined whether there is state representative data for each NFHS survey in Table S1. The legend below classifies each state for each NFHS survey into one of three possible categories: state representative data is available, districts were re-assigned to that state or removed from that state, or there is no data available at all.

© 2023 Rajpal S et al. *JAMA Network Open.*

eTable 1. Overview of Data Comparability for NFHS Round by State or Union Territory in India

States and Union Territories (2021)	NFHS-1	NFHS-2	NFHS-3	NFHS-4	NFHS-5
Andhra Pradesh	⚠	⚠	⚠	✓	✓
Arunachal Pradesh	✓	✓	✓	✓	✓
Assam	✓	✓	✓	✓	✓
Bihar	⚠	⚠	✓	✓	✓
Chhattisgarh	⚠	⚠	✓	✓	✓
Goa	✓	✓	✓	✓	✓
Gujarat	✓	✓	✓	✓	✓
Haryana	✓	✓	✓	✓	✓
Himachal Pradesh	✓	✓	✓	✓	✓
Jharkhand	⚠	⚠	✓	✓	✓
Karnataka	✓	✓	✓	✓	✓
Kerala	✓	✓	✓	✓	✓
Madhya Pradesh	⚠	⚠	✓	✓	✓
Maharashtra	✓	✓	✓	✓	✓
Manipur	✓	✓	✓	✓	✓
Meghalaya	✓	✓	✓	✓	✓
Mizoram	✓	✓	✓	✓	✓
Nagaland	✓	✓	✓	✓	✓
Odisha	✓	✓	✓	✓	✓
Punjab	✓	✓	✓	✓	✓
Rajasthan	✓	✓	✓	✓	✓
Sikkim	✗	✓	✓	✓	✓
Tamil Nadu	✓	✓	✓	✓	✓
Telangana	⚠	⚠	⚠	✓	✓
Tripura	✓	✓	✓	✓	✓
Uttar Pradesh	⚠	⚠	✓	✓	✓
Uttarakhand	⚠	⚠	✓	✓	✓
West Bengal	✓	✓	✓	✓	✓
Andaman & Nicobar Island (UT)	✗	✗	✗	✓	✓
Chandigarh (UT)	✗	✗	✗	✓	✓
Dadra Nagar Haveli & Daman & Diu (UT)	✗	✗	✗	✓	✓
NCT Delhi (UT)	✓	✓	✓	✓	✓
Jammu & Kashmir (UT)	⚠	⚠	⚠	⚠	✓
Ladakh (UT)	✗	✗	⚠	⚠	✓

(✓ = State-comparable data available (direct comparability); ⚠ = State-comparable data not available and districts reassigned for comparability (indirect comparability); ✗ = Data not available)

© 2023 Rajpal S et al. *JAMA Network Open.*

eTable 2. Summary of the Method Used for Creating State Equivalence for Each NFHS Survey

NFHS Survey and Time Period	State Equivalence Method Summary
NFHS-1: 1992-1993	Districts were re-assigned to the current state and parent state.
NFHS-2: 1998-1998	Districts were re-assigned to the current state and parent.
NFHS-3: 2005-2006	No district data is available, and pre-existing state value is applied to the parent and new state when a new state was formed.
NFHS-4: 2015-2016	Kargil and Leh districts from Jammu and Kashmir were assigned to the new union territory of Ladakh. Dadra and Nagar Haveli and Daman and Diu were merged into one state.
NFHS-5: 2019-2021	No changes were made.

© 2023 Rajpal S et al. *JAMA Network Open*.

eTable 3. Number of Districts From NFHS-1 and NFHS-2 That Fall Into the Current States

States and Union Territories (2021)	NFHS-1	NFHS-2
Andhra Pradesh†	13	13
Arunachal Pradesh	9	11
Assam	20	20
Bihar†	26	29
Chhattisgarh†	7	7
Goa	1	2
Gujarat	18	19
Haryana	12	16
Himachal Pradesh	11	11
Jharkhand†	4	12
Karnataka	19	20
Kerala	12	14
Madhya Pradesh†	36	37
Maharashtra	25	28
Manipur	5	8
Meghalaya	5	5
Mizoram	3	3
Nagaland	7	7
Odisha	13	13
Punjab	12	12
Rajasthan	25	27
Sikkim	0	4
Tamil Nadu	17	21
Telangana†	9	10
Tripura	3	3
Uttar Pradesh†	46	53
Uttarakhand†	8	8
West Bengal	16	17
Andaman & Nicobar Island (UT)	0	0
Chandigarh (UT)	0	0
Dadra Nagar Haveli & Daman & Diu (UT)	0	0
NCT Delhi (UT)	1	1
Jammu & Kashmir (UT) †	6	9
Ladakh (UT) †	0	0
Lakshadweep (UT)	0	0
Puducherry (UT)	0	0
**Districts not Assigned to any State**	3	0

†These marked states are not state representative, as they have had districts added or removed from them.

© 2023 Rajpal S et al. *JAMA Network Open.*

eAppendix 3. Statistical Analysis: Annual Absolute and Relative Change

We computed the annual absolute change (AAC) in the prevalence of zero-dose children. The computation of AAC (% points per annum) between two survey points was done using the following equation:

$$\text{AAC (\% points per annum)} = (P_t - P_{t-n}) * (\frac{1}{n}).$$

Here, P_t is prevalence (%) at time t, P_{t-n} is prevalence (%) at the time before *n* years from time t, and n refers to the number of years between two survey points. This way, a negative (positive) AAC value depicts a reduction (increase) in the prevalence, respectively.

Since the base prevalence varies across states, we also computed relative change (%) for all states using the formula:

$$\text{ARC (\%)} = (P_t - P_{t-n})/(P_t) * (\frac{1}{n}).$$

© 2023 Rajpal S et al. *JAMA Network Open*.

eAppendix 4. Statistical Analysis : Variance Partitioning: 4-Level Regression Model

For each of the five surveys (except for NFHS-3, 2006), we employed a four-level logistic regression model to partition the total variation in the prevalence of zero-dose children (Y) across children i (level-1); cluster j (level-2); district k (level-3); and state l (level-4):

$$Y_{ijkl} = \beta_0 + (u_{0jkl} + v_{0kl} + f_{0l}).$$

In the model mentioned above, u_{0jkl}, v_{0kl}, f_{0l} are model residuals specific to cluster, district, and state, respectively. These residuals are assumed to have a normal distribution with a mean of 0 and variance of $u_{0jkl} \sim (0,\ \sigma^2_{u0})$; $v_{0kl} \sim (0,\ \sigma^2_{v0})$; $f_{0l} \sim (0,\ \sigma^2_{f0})$. Here, the term σ^2_{u0} denotes within-district, inter-cluster variation, σ^2_{v0} denotes within-state, inter-district variation and σ^2_{f0} stands for inter-state variation. For binary outcomes, variance across individual children is not computed directly and is instead assumed to follow a logistic distribution with a fixed variance of $\pi^2/3$ or 3.29. We then computed the variance partitioning coefficient to assess the significance of each geographical unit (z) in total variability as follows:

$$\left(\frac{\sigma^2_z}{\sigma^2_{u0} + \sigma^2_{v0} + \sigma^2_{f0}}\right) * 100.$$

Multilevel regression analysis was not performed for NFHS-3 (2006) because of the non-availability of district identifiers in the sample design. Multilevel modeling was performed using the STATA 15 and MLwiN 3.0 software program (using Stata's runmlwin command) and the Monte Carlo Markov Chain (MCMC) method using the Gibbs sampler, keeping the default prior distribution of Iterated Generalized Least Square (IGLS) as the starting value.

© 2023 Rajpal S et al. *JAMA Network Open.*

eFigure 2. Estimated Count of 0-Dose Children (Aged 12-23 Months) by States in India, NFHS 2021

Supplementary Main Analysis – Zero-dose coded as non-receipt of DTP1

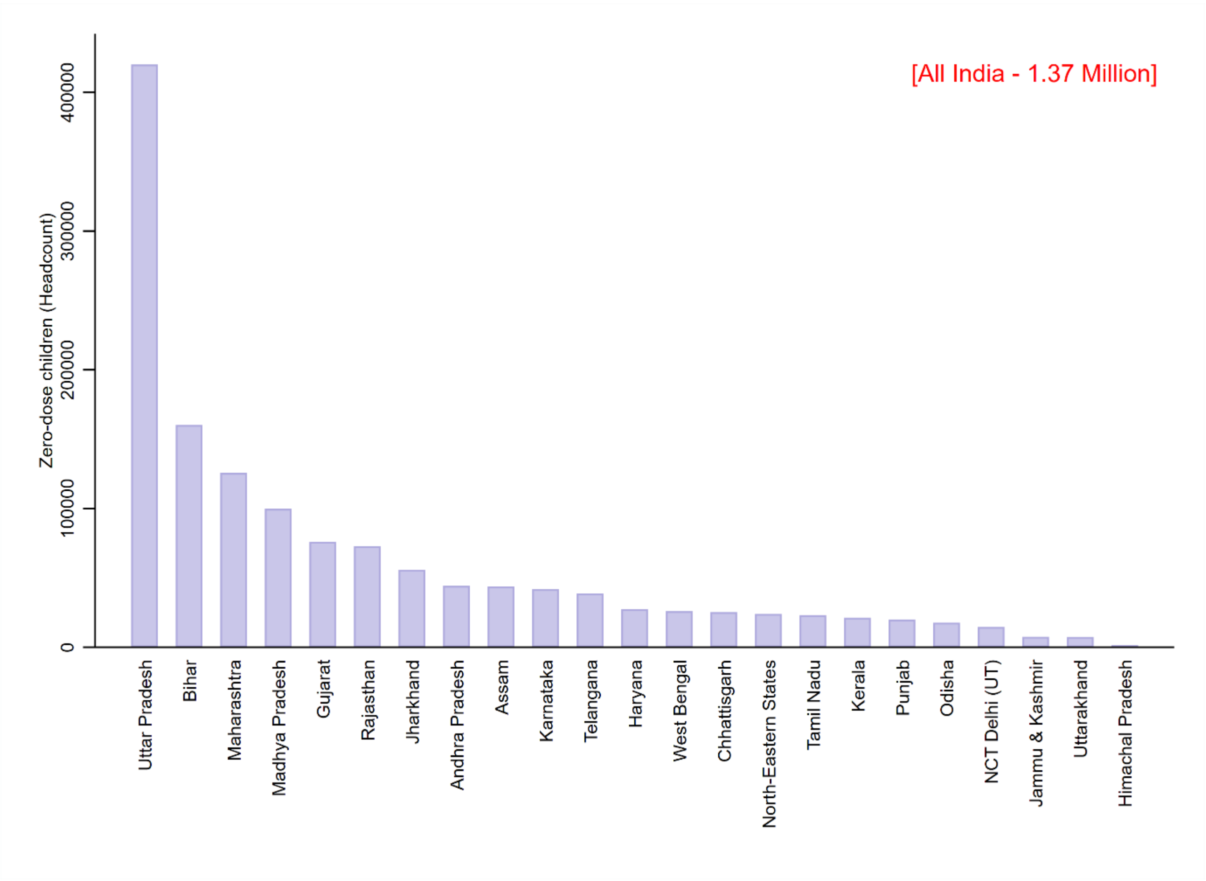

Note: North-Eastern states include Tripura, Meghalaya, Nagaland, Mizoram, Manipur, Sikkim

© 2023 Rajpal S et al. *JAMA Network Open*.

eTable 4. Prevalence of 0-Dose Children (Aged 12-23 Months) by States and Union Territories and Rural and Urban Areas in India, NFHS 1993-2021

State	1993		1999		2006		2016		2021	
	Rural	Urban	Rural	Urban	Rural	Urban	Rural	Urban	Rural	Urban
India	**37.6**	**19.2**	**30.4**	**12.5**	**26.5**	**15.4**	**10.6**	**9.4**	**6.3**	**7.5**
Andhra Pradesh	21.3	17.9	11.1	8.4	4.9	6.5	3.6	1.5	4.7	10.4
Arunachal Pradesh	53.4	30.8	46.1	26.3	44.0	31.7	31.6	20.4	14.7	7.6
Assam	49.3	17.6	42.1	13.8	32.8	24.6	18.8	5.7	10.3	6.9
Bihar	57.5	46.0	60.9	52.5	34.3	38.5	9.8	9.0	6.3	6.7
Chhattisgarh	38.7	7.0	38.0	11.8	12.6	8.1	2.0	1.5	3.6	9.3
Goa	5.6	6.7	0.0	4.5	1.9	3.1	0.0	6.0	0.0	3.1
Gujarat	24.9	15.9	17.2	14.4	21.0	14.3	16.4	11.8	7.6	7.5
Haryana	21.1	13.3	11.4	7.6	18.7	6.7	10.7	13.1	6.4	6.3
Himachal Pradesh	10.5	2.2	3.4	2.8	3.6	2.0	3.3	7.8	1.3	2.2
Jharkhand	71.4	25.0	60.1	33.2	38.5	16.8	6.8	4.6	6.8	14.3
Karnataka	18.9	20.0	13.2	9.6	16.5	8.3	7.3	14.0	4.2	5.7
Kerala	16.5	9.4	5.2	1.8	5.6	2.8	4.1	3.9	6.1	4.8
Madhya Pradesh	44.0	25.8	44.6	18.8	26.7	13.1	11.4	5.8	6.7	4.9
Maharashtra	8.9	10.1	5.4	2.0	8.5	3.4	14.0	14.9	6.5	10.8
Manipur	42.4	16.7	24.2	21.7	26.8	11.4	11.8	5.0	7.8	3.7
Meghalaya	66.9	44.0	57.4	39.3	38.6	29.7	17.5	7.0	16.9	19.0
Mizoram	22.8	9.4	18.1	2.7	17.3	2.9	27.9	20.0	12.6	17.4
Nagaland	82.8	57.7	55.7	45.0	55.7	38.0	35.3	24.3	19.3	11.6
Odisha	31.2	29.4	18.5	20.3	15.3	20.0	6.3	7.2	3.2	0.8
Punjab	20.6	9.8	15.5	2.4	14.8	8.3	2.2	4.3	4.9	7.2
Rajasthan	56.4	28.4	52.4	38.6	37.6	24.1	15.8	6.8	5.9	3.8
Sikkim			24.0	15.4	5.6	2.4	0.0	1.8	6.3	0.0
Tamil Nadu	5.6	3.3	1.7	0.0	0.7	1.5	8.5	6.6	2.4	3.4
Telangana	29.5	11.3	11.6	12.4			2.9	1.3	5.7	10.2
Tripura	44.9	28.6	33.8	0.0	20.2	13.0	18.6	10.1	5.5	4.8
Uttar Pradesh	51.1	33.7	44.0	19.9	45.2	38.2	16.5	16.2	9.3	11.7
Uttarakhand	35.1	10.3	10.7	15.0	16.3	19.4	8.6	7.4	4.3	5.1
West Bengal	29.9	15.0	25.1	9.3	9.9	9.2	1.7	7.6	2.5	3.6
Union Territories										
NCT Delhi	9.3	10.3	8.1	7.7	15.8	16.2	19.4	6.1	0.0	5.9
Jammu & Kashmir	17.8	6.1	13.9	4.3	10.9	3.2	6.3	3.8	4.1	9.1
Andaman & Nicobar							9.7	18.5	3.4	0.0
Chandigarh							0.0	4.6	0.0	6.4
Dadra & n; D & Diu							10.4	13.6	1.2	4.7
Lakshadweep							0.0	4.0	14.1	6.3
Ladakh							2.0	0.0	0.0	3.8
Puducherry							1.2	0.1	1.4	0.8

© 2023 Rajpal S et al. *JAMA Network Open*.

eTable 5. Annual Relative Percentage Change (Percentage) in 0-Dose Children Across States and Union Territories in India, NFHS 1993-2021

State	1993-1999	1999-2006	2006-2016	2016-2021	1993-2021
All India	-3.04	-1.47	-5.64	-7.38	-2.78
Andhra Pradesh	-6.90	-6.80	-4.55	24.67	-2.31
Arunachal Pradesh	-2.13	-0.07	-2.99	-11.46	-2.57
Assam	-1.84	-4.55	-3.88	-10.65	-2.86
Bihar	1.12	-6.05	-7.21	-6.80	-3.05
Chhattisgarh	-0.84	-9.28	-8.39	30.53	-2.99
Goa	-10.30	12.61	4.69	-5.11	-1.47
Gujarat	-3.72	2.20	-2.19	-10.41	-2.35
Haryana	-6.51	7.35	-2.70	-9.66	-2.38
Himachal Pradesh	-9.52	0.43	0.88	-11.89	-2.93
Jammu & Kashmir	-3.59	2.22	-4.89	-5.83	-2.37
Jharkhand	-1.87	-5.51	-8.11	4.69	-3.02
Karnataka	-5.33	1.42	-2.41	-10.69	-2.61
Kerala	-9.94	-1.90	-0.26	7.89	-2.21
Madhya Pradesh	-0.47	-5.50	-5.79	-7.47	-2.91
Maharashtra	-7.99	6.62	13.83	-8.81	-0.48
Manipur	-4.38	0.91	-6.12	-5.57	-2.74
Meghalaya	-1.70	-4.77	-5.37	-0.12	-2.52
Mizoram	-4.18	-1.60	13.11	-7.98	-0.44
Nagaland	-4.48	-0.48	-3.80	-10.06	-2.74
Odisha	-5.69	-2.00	-5.94	-11.08	-3.12
Punjab	-4.97	1.09	-7.64	16.67	-2.40
Rajasthan	-0.66	-4.17	-6.05	-12.03	-3.08
Tamil Nadu	-11.01	0.00	60.00	-13.51	-1.65
Tripura	-4.68	-4.60	-0.51	-14.84	-3.06
Uttar Pradesh	-2.47	1.32	-6.26	-8.17	-2.76
Uttarakhand	-8.29	6.59	-5.15	-8.67	-2.87
West Bengal	-2.26	-8.55	-6.56	-6.45	-3.18
Telangana	-7.32			58.00	-2.34
Sikkim		-11.12	-8.82	153.33	
Union Territory					
NCT Delhi (UT)	-3.36	15.20	-6.09	-3.17	-1.66
Andaman & Nicobar				-16.88	
Chandigarh				12.20	
Dadra & n; Daman				-15.96	
Lakshadweep				25.63	
Ladakh				-20.00	
Puducherry				16.00	

Note Negative and positive values depict decline and rise, respectively.

© 2023 Rajpal S et al. *JAMA Network Open.*

eTable 6. Annual Absolute Change (Percentage Points) in the Prevalence of 0-Dose Children by States in India, NFHS 1993-2021

State	1993-1999	1999-2006	2006-2016	2016-2021	1993-2021
All India	-1.01	-0.39	-1.33	-0.76	-0.93
Andhra Pradesh	-1.40	-0.71	-0.25	0.74	-0.47
Arunachal Pradesh	-1.06	-0.03	-1.26	-3.38	-1.28
Assam	-0.86	-1.84	-1.07	-1.80	-1.33
Bihar	0.63	-3.66	-2.51	-0.66	-1.71
Chhattisgarh	-0.30	-3.13	-0.99	0.58	-1.07
Goa	-0.63	0.21	0.15	-0.24	-0.09
Gujarat	-0.81	0.36	-0.41	-1.52	-0.51
Haryana	-1.26	0.77	-0.43	-1.12	-0.46
Himachal Pradesh	-0.94	0.01	0.03	-0.44	-0.29
Jammu & Kashmir	-0.59	0.27	-0.69	-0.42	-0.39
Jharkhand	-1.19	-3.03	-2.74	0.30	-1.91
Karnataka	-1.03	0.17	-0.32	-1.08	-0.50
Kerala	-1.47	-0.09	-0.01	0.30	-0.33
Madhya Pradesh	-0.19	-2.10	-1.36	-0.74	-1.15
Maharashtra	-0.74	0.27	0.83	-1.26	-0.04
Manipur	-1.49	0.21	-1.53	-0.54	-0.93
Meghalaya	-1.07	-2.64	-1.98	-0.02	-1.58
Mizoram	-0.69	-0.19	1.35	-1.90	-0.07
Nagaland	-3.53	-0.26	-1.99	-3.26	-2.16
Odisha	-1.76	-0.37	-0.95	-0.72	-0.97
Punjab	-0.90	0.13	-0.97	0.50	-0.43
Rajasthan	-0.34	-2.06	-2.11	-1.66	-1.59
Tamil Nadu	-0.53	0.00	0.66	-1.04	-0.08
Tripura	-2.01	-1.33	-0.10	-2.76	-1.32
Uttar Pradesh	-1.20	0.53	-2.74	-1.34	-1.34
Uttarakhand	-2.31	0.77	-0.88	-0.72	-0.80
West Bengal	-0.60	-1.91	-0.59	-0.20	-0.84
Telangana	-1.77			1.16	-0.57
Sikkim		-2.56	-0.45	0.92	
Union Territories					
NCT Delhi (UT)	-0.34	1.19	-0.98	-0.20	-0.17
Andaman & Nicobar				-2.38	
Chandigarh				0.50	
Dadra & n; Daman				-1.74	
Lakshadweep				0.82	
Ladakh				-0.30	
Puducherry				0.08	

Note: Negative and positive values depict decline and rise, respectively.

© 2023 Rajpal S et al. *JAMA Network Open.*

eTable 7. Annual Absolute Change (Percentage Points) in 0-Dose Children Across States and Union Territories and Rural and Urban Areas in India, NFHS 1993-2021

	1993-1999		1999-2006		2006-2016		2016-2021		1993-2021	
**State**	Rural	Urban	Rural	Urban	Rural	Urban	Rural	Urban	Rural	Urban
**India**	-1.02	-0.95	-0.55	0.41	-1.59	-0.60	-0.86	-0.38	-1.07	-0.40
Andhra Pradesh	-1.46	-1.36	-0.89	-0.27	-0.13	-0.50	0.22	1.78	-0.57	-0.26
Arunachal Pradesh	-1.04	-0.64	-0.30	0.77	-1.24	-1.13	-3.38	-2.56	-1.33	-0.80
Assam	-1.03	-0.54	-1.33	1.54	-1.40	-1.89	-1.70	0.24	-1.34	-0.37
Bihar	0.49	0.93	-3.80	-2.00	-2.45	-2.95	-0.70	-0.46	-1.77	-1.36
Chhattisgarh	-0.10	0.69	-3.63	-0.53	-1.06	-0.66	0.32	1.56	-1.21	0.08
Goa	-0.80	-0.31	0.27	-0.20	-0.19	0.29	0.00	-0.58	-0.19	-0.12
Gujarat	-1.10	-0.21	0.54	-0.01	-0.46	-0.25	-1.76	-0.86	-0.60	-0.29
Haryana	-1.39	-0.81	1.04	-0.13	-0.80	0.64	-0.86	-1.36	-0.51	-0.24
Himachal Pradesh	-1.01	0.09	0.03	-0.11	-0.03	0.58	-0.40	-1.12	-0.32	0.00
Jammu & Kashmir	-0.56	-0.26	-0.43	-0.16	-0.46	0.06	-0.44	1.06	-0.47	0.10
Jharkhand	-1.61	1.17	-3.09	-2.34	-3.17	-1.22	0.00	1.94	-2.23	-0.37
Karnataka	-0.81	-1.49	0.47	-0.19	-0.92	0.57	-0.62	-1.66	-0.51	-0.49
Kerala	-1.61	-1.09	0.06	0.14	-0.15	0.11	0.40	0.18	-0.36	-0.16
Madhya Pradesh	0.09	-1.00	-2.56	-0.81	-1.53	-0.73	-0.94	-0.18	-1.29	-0.72
Maharashtra	-0.50	-1.16	0.44	0.20	0.55	1.15	-1.50	-0.82	-0.08	0.02
Manipur	-2.60	0.71	0.37	-1.47	-1.50	-0.64	-0.80	-0.26	-1.19	-0.45
Meghalaya	-1.36	-0.67	-2.69	-1.37	-2.11	-2.27	-0.12	2.40	-1.72	-0.86
Mizoram	-0.67	-0.96	-0.11	0.03	1.06	1.71	-3.06	-0.52	-0.35	0.28
Nagaland	-3.87	-1.81	0.00	-1.00	-2.04	-1.37	-3.20	-2.54	-2.19	-1.59
Odisha	-1.81	-1.30	-0.46	-0.04	-0.90	-1.28	-0.62	-1.28	-0.97	-0.99
Punjab	-0.73	-1.06	-0.10	0.84	-1.26	-0.40	0.54	0.58	-0.54	-0.09
Rajasthan	-0.57	1.46	-2.11	-2.07	-2.18	-1.73	-1.98	-0.60	-1.74	-0.85
Tamil Nadu	-0.56	-0.47	-0.14	0.21	0.78	0.51	-1.22	-0.64	-0.11	0.00
Telangana	-2.56	0.16					0.56	1.78	-0.82	-0.04
Tripura	-1.59	-4.09	-1.94	1.86	-0.16	-0.29	-2.62	-1.06	-1.36	-0.82
Uttar Pradesh	-1.01	-1.97	0.17	2.61	-2.87	-2.20	-1.44	-0.90	-1.44	-0.76
Uttarakhand	-3.49	0.67	0.80	0.63	-0.77	-1.20	-0.86	-0.46	-1.06	-0.18
West Bengal	-0.69	-0.81	-2.17	-0.01	-0.82	-0.16	0.16	-0.80	-0.94	-0.39
Sikkim			-2.63	-1.86	-0.56	-0.06	1.26	-0.36		
Union Territories										
NCT Delhi	-0.17	-0.37	1.10	1.21	0.36	-1.01	-3.88	-0.04	-0.32	-0.15
Andaman & Nicobar							-1.26	-3.70		
Chandigarh							0.00	0.36		
Dadra & n; D & Diu							-1.84	-1.78		
Lakshadweep							2.82	0.46		
Ladakh							-0.40	0.76		
Puducherry							0.04	0.14		

Note Negative and Positive values depict decline and rise, respectively.

© 2023 Rajpal S et al. *JAMA Network Open*.

eTable 8. Annual Relative Change (Percentage) in 0-Dose Children Across States and Union Territories and Rural and Urban Areas in India, NFHS 1993-2021

	1993-1999		1999-2006		2006-2016		2016-2021		1993-2021	
**State**	Rural	Urban	Rural	Urban	Rural	Urban	Rural	Urban	Rural	Urban
**India**	-2.73	-4.98	-1.83	3.31	-6.0	-3.89	-8.11	-4.04	-2.87	-2.10
Andhra Pradesh	-6.84	-7.58	-7.98	-3.23	-2.65	-7.69	6.11	118.67	-2.69	-1.44
Arunachal Pradesh	-1.95	-2.09	-0.65	2.93	-2.82	-3.56	-10.70	-12.55	-2.50	-2.60
Assam	-2.09	-3.08	-3.16	11.18	-4.27	-7.68	-9.04	4.21	-2.73	-2.10
Bihar	0.84	2.02	-6.24	-3.81	-7.14	-7.66	-7.14	-5.11	-3.07	-2.95
Chhattisgarh	-0.26	9.80	-9.55	-4.48	-8.41	-8.15	16.00	104.00	-3.13	1.13
Goa	-14.29	-4.69		-4.44	-10.00	9.35		-9.67	-3.45	-1.85
Gujarat	-4.42	-1.35	3.16	-0.10	-2.19	-1.75	-10.73	-7.29	-2.40	-1.82
Haryana	-6.57	-6.12	9.15	-1.69	-4.28	9.55	-8.04	-10.38	-2.40	-1.81
Himachal Pradesh	-9.66	3.90	0.84	-4.08	-0.83	29.00	-12.12	-14.36	-3.02	0.00
Jammu & Kashmir	-3.13	-4.22	-3.08	-3.65	-4.22	1.88	-6.98	27.89	-2.65	1.70
Jharkhand	-2.26	4.69	-5.13	-7.06	-8.23	-7.26	0.00	42.17	-3.12	-1.48
Karnataka	-4.31	-7.43	3.57	-1.93	-5.58	6.87	-8.49	-11.86	-2.68	-2.47
Kerala	-9.78	-11.55	1.10	7.94	-2.68	3.93	9.76	4.62	-2.17	-1.69
Madhya Pradesh	0.19	-3.88	-5.73	-4.33	-5.73	-5.57	-8.25	-3.10	-2.92	-2.79
Maharashtra	-5.62	-11.46	8.20	10.00	6.47	33.82	-10.71	-5.50	-0.93	0.24
Manipur	-6.13	4.28	1.53	-6.78	-5.60	-5.61	-6.78	-5.20	-2.81	-2.68
Meghalaya	-2.03	-1.53	-4.68	-3.49	-5.47	-7.64	-0.69	34.29	-2.58	-1.96
Mizoram	-2.94	-10.18	-0.63	1.06	6.13	58.97	-10.97	-2.60	-1.54	2.93
Nagaland	-4.68	-3.14	0.00	-2.22	-3.66	-3.61	-9.07	-10.45	-2.64	-2.76
Odisha	-5.82	-4.42	-2.47	-0.21	-5.88	-6.40	-9.84	-17.78	-3.09	-3.35
Punjab	-3.54	-10.79	-0.65	35.12	-8.51	-4.82	24.55	13.49	-2.63	-0.91
Rajasthan	-1.01	5.13	-4.03	-5.37	-5.80	-7.18	-12.53	-8.82	-3.09	-2.99
Tamil Nadu	-9.95	-14.29	-8.40		111.43	34.00	-14.35	-9.70	-1.97	0.10
Telangana	-8.67	1.39					19.31	136.92	-2.78	-0.34
Tripura	-3.53	-14.29	-5.75		-0.79	-2.23	-14.09	-10.50	-3.03	-2.87
Uttar Pradesh	-1.98	-5.85	0.39	13.14	-6.35	-5.76	-8.73	-5.56	-2.82	-2.25
Uttarakhand	-9.93	6.52	7.48	4.19	-4.72	-6.19	-10.00	-6.22	-3.03	-1.74
West Bengal	-2.29	-5.43	-8.65	-0.15	-8.28	-1.74	9.41	-10.53	-3.16	-2.62
Sikkim			-10.95	-12.06	-10.00	-2.50		-20.00		
Union Territories										
NCT Delhi	-1.84	-3.61	13.58	15.77	2.28	-6.23	-20.00	-0.66	-3.45	-1.47
Andaman & Nicobar							-12.99	-20.00		
Chandigarh								7.83		
Dadra & n; D & Diu							-17.69	-13.09		
Lakshadweep								11.50		
Ladakh							-20.00			
Puducherry							3.33	140.00		

Note: Negative and positive values depict decline and rise, respectively.

© 2023 Rajpal S et al. *JAMA Network Open*.

eTable 9. Distribution (Percentage) of 0-Dose Children Across States and Union Territories in India, NFHS 1993-2021

State	1993		1999		2006		2016		2021	
	Population Proportion (%)	Zero-dose (%)	Population Proportion (%)	Zero-dose (%)	Population Proportion (%)	Zero-dose (%)	Population Proportion (%)	Zero-dose (%)	Population Proportion (%)	Zero-dose (%)
**India**	100	100	100	100	100	100	100	100	100	100
Andhra Pradesh	2.05	02.03	2.08	01.70	4.5	01.30	1.16	01.05	1.23	03.50
Arunachal Pradesh	1.38	00.21	1.27	00.16	1.59	00.20	1.74	00.21	2.21	00.13
Assam	3.51	04.12	3.33	03.52	2.7	02.26	3.92	03.21	4.53	01.83
Bihar	6.77	16.94	7.11	21.02	4.63	17.17	9.94	12.26	9.23	13.35
Chhattisgarh	1.34	02.17	1.09	02.69	2.89	01.00	3.14	00.42	3.73	01.68
Goa	2.41	00.02	1.3	00.01	2.16	00.01	0.18	00.04	0.16	00.03
Gujarat	4.08	03.01	3.93	02.82	3.22	03.94	2.86	05.94	4.36	04.54
Haryana	3.91	01.39	3.5	00.84	2.4	01.24	3.07	02.63	3.02	01.82
Himachal Pradesh	2.93	00.16	3.03	00.07	2	00.07	1.15	00.17	1.23	00.11
Jammu & Kashmir (UT)	3.18	00.22	3.23	00.41	2.6	00.31	2.98	00.36	2.30	00.46
Jharkhand	0.69	02.56	1.79	05.08	3.12	04.63	4.88	01.99	4.46	03.96
Karnataka	4.73	02.90	4.34	02.24	4.29	02.79	3.01	03.90	3.66	03.24
Kerala	3.41	01.05	2.49	00.48	2.27	00.29	1	00.63	1.17	01.46
Madhya Pradesh	6.29	07.69	7.63	09.63	5.5	06.51	9.28	06.46	7.07	06.00
Maharashtra	4.29	02.41	5.81	01.55	6.38	02.22	3.6	11.67	4.23	09.82
Manipur	1.1	00.23	2.15	00.23	3.53	00.20	2.35	00.19	1.34	00.19
Meghalaya	1.24	00.39	1.75	00.62	2.16	00.51	1.72	00.49	2.64	00.91
Mizoram	0.95	00.03	1.7	00.05	1.6	00.04	2.03	00.21	1.10	00.18
Nagaland	1.39	00.38	1.43	00.44	4.42	00.42	1.82	00.42	1.31	00.22
Odisha	4.38	03.14	4.8	02.51	3.51	02.40	4.29	02.06	3.60	01.40
Punjab	3.03	01.16	2.71	00.86	2.47	01.06	2.09	00.50	2.39	01.55
Rajasthan	5.6	07.72	8.29	11.12	3.82	09.18	6.43	08.08	5.78	05.15
Sikkim		-	1.49	0.05	1.38	00.01	0.42	00.00	0.27	00.02
Tamil Nadu	3.64	00.76	4.6	0.26	3.26	00.16	3.18	04.46	2.98	01.98
Telangana	1.58	01.86	2	1.81		-	0.97	00.57	3.38	02.83
Tripura	1.05	00.36	1	00.03	1.24	00.24	0.5	00.34	0.90	00.16
Uttar Pradesh	14.52	30.47	8.66	21.99	13.23	38.32	15.77	28.36	15.01	29.86
Uttarakhand	2.13	00.69	0.72	00.30	2.27	00.52	2.24	00.64	1.62	00.58
West Bengal	4.43	05.56	4.13	6.86	4.6	02.38	2.26	01.86	2.50	01.82
Union Territories										
NCT Delhi	3.98	00.36	2.65	0.36	2.28	00.60	0.62	00.77	1.32	01.08
Andaman & Nicobar		-		-		-	0.27	00.03	0.21	00.01
Chandigarh		-		-		-	0.08	00.02	0.06	00.06
Dadra & n; Daman & D		-		-		-	0.29	00.04	0.35	00.01
Puducherry		-		-		-	0.12	00.01	0.10	00.01
Lakshadweep		-		-		-	0.26	00.00	0.19	00.01
Ladakh		-		-		-	0.39	00.01	0.35	00.01

© 2023 Rajpal S et al. *JAMA Network Open*.

eFigure 3. Maps for the Prevalence of 0-Dose Children (Aged 12-23 Months) by State in India, NFHS 1993-2021

(A) 1993 (B) 1999 (C) 2006 (D) 2016 (E) 2021

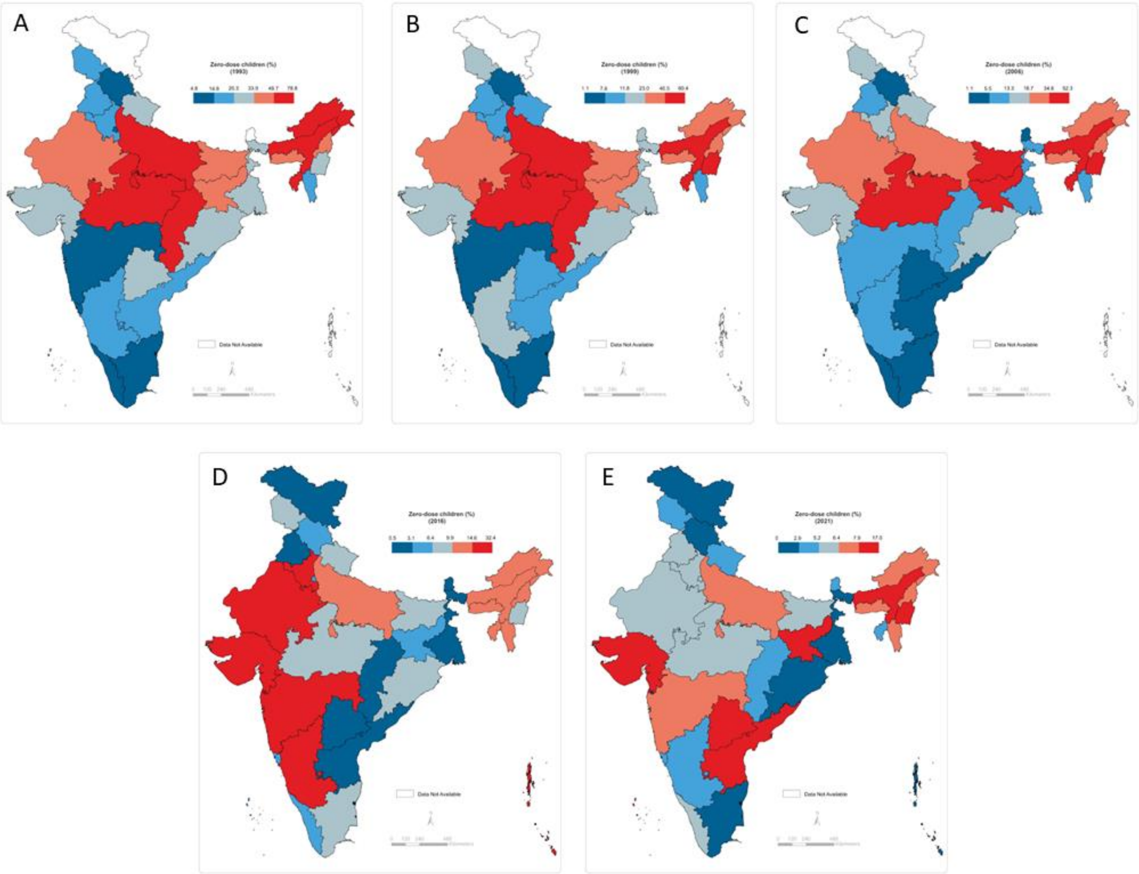

© 2023 Rajpal S et al. *JAMA Network Open*.

eFigure 4. Maps for Annual Absolute Change (Percentage Points per Year) in the Prevalence of 0-Dose Children Across States and Union Territories in India, NFHS 1993-2021

(A) 2016-2021

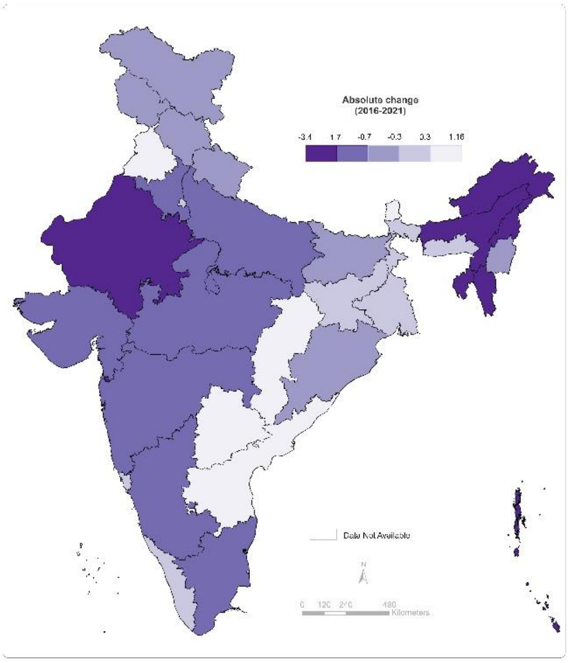

(B) 1993-2021

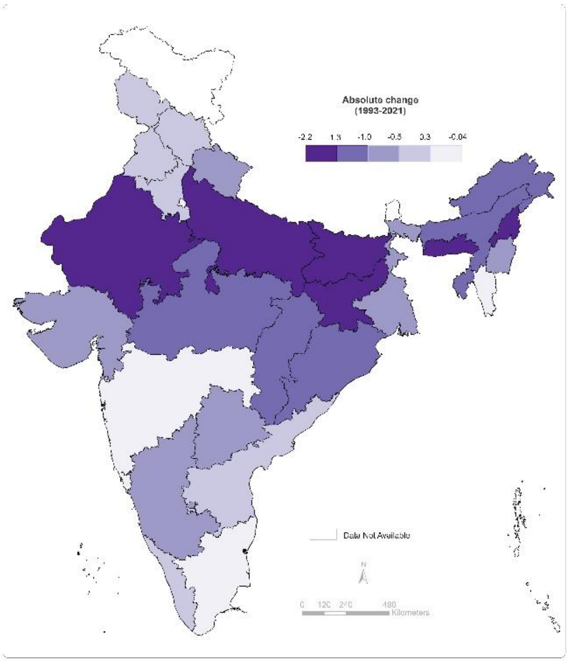

© 2023 Rajpal S et al. *JAMA Network Open*.

eFigure 5. Panel-Line Plots for the Prevalence of 0-Dose Children (aged 12-23 Months) Across States, Rural and Urban Areas, and All India, NFHS 1993-2021

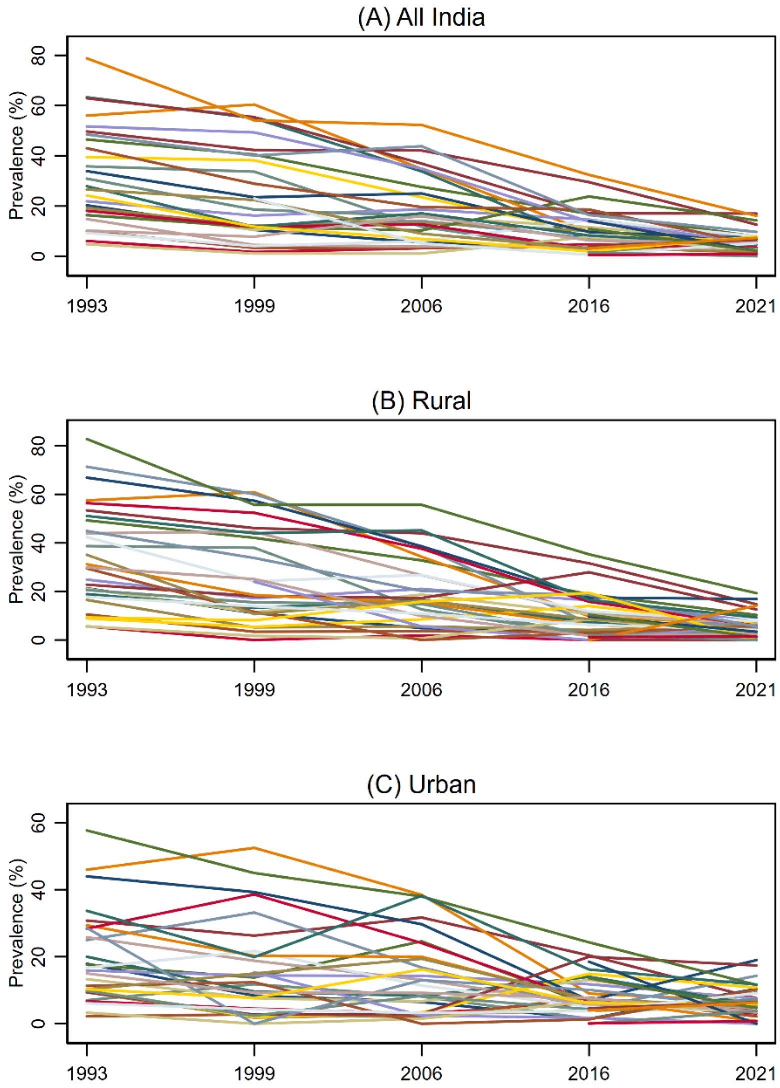

© 2023 Rajpal S et al. *JAMA Network Open*.

eFigure 6. Scatter Plot for Correlation Between Baseline Prevalence and Annual Absolute Change (Percentage Points) in the Prevalence of 0-Dose Children in India, NFHS 1993-2021

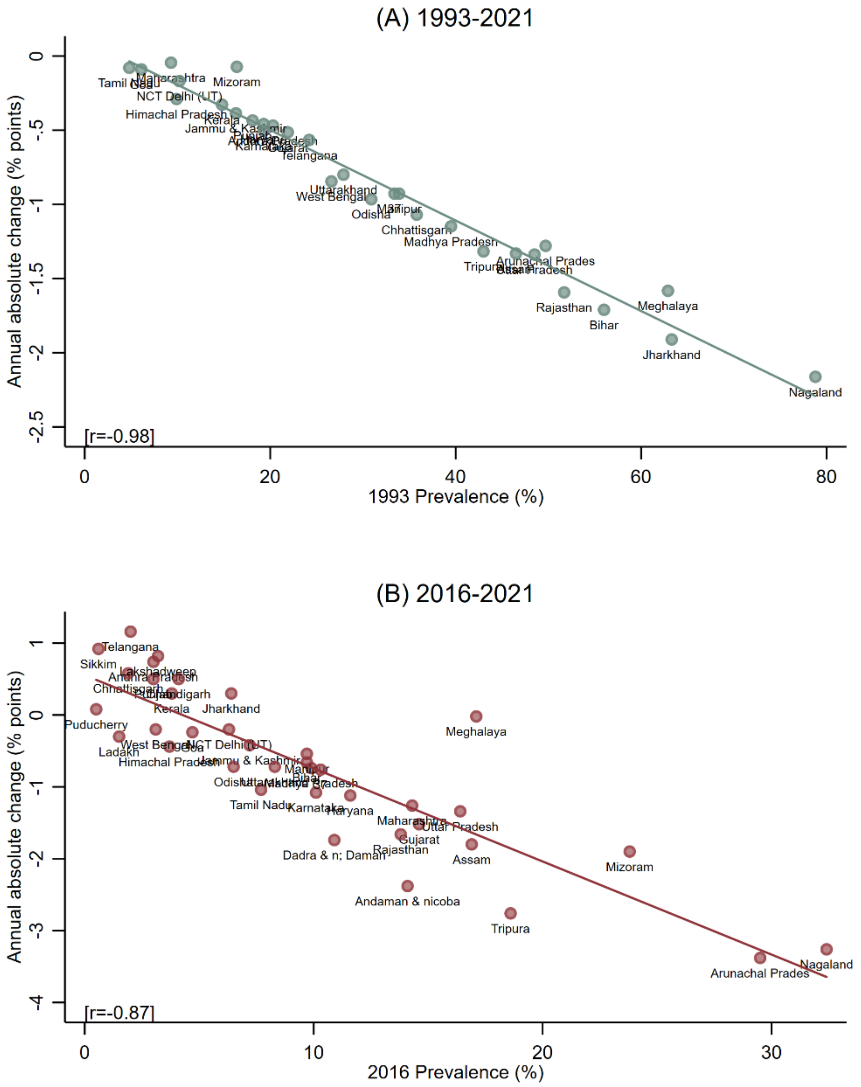

Note: Negative and positive values depict decrease and increase, respectively.

© 2023 Rajpal S et al. *JAMA Network Open*.

eFigure 7. Scatter Plot for Correlation Between Baseline Prevalence and Annual Absolute Change (Percentage Points) in the Prevalence of 0-Dose Children in India, NFHS 1992-2021

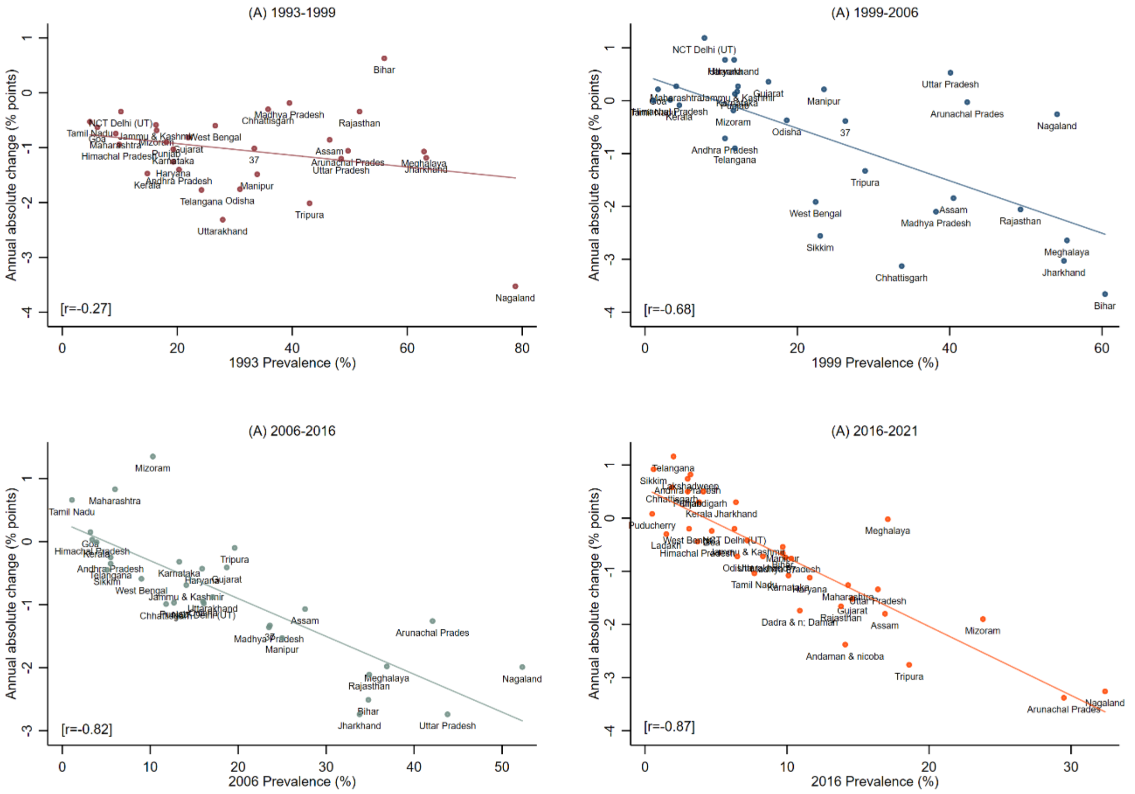

Note: Negative and positive values depict decrease and increase, respectively.

© 2023 Rajpal S et al. *JAMA Network Open*.

eTable 10. Variance Partition Coefficients (VPCs) (Percentage) in 0-Dose Children by Multiple Geographic Regions in India, NFHS 1993-2021

Year	1993		1999		2016		2021	
Geographies	Var	SE	Var	SE	Var	SE	Var	SE
State	1.71	0.60	2.47	0.81	1.04	0.32	0.38	0.12
District	0.42	0.07	0.32	0.07	0.62	0.05	0.28	0.04
Cluster	0.8	0.08	1.11	0.13	1.34	0.07	1.19	0.07

Note: Estimates derived from four-level logistic regression model were adjusted for state, district, and cluster random effects. VPC for 2006 (NFHS-3) was not estimated due to non-availability of district identifiers in the data.

© 2023 Rajpal S et al. *JAMA Network Open.*

eTable 11. Mean Probability of 0-Dose Children With 95% Coverage Boundaries Across Geographic Regions in India, NFHS 1993-2021

Year	1993		1999		2016		2021	
Geographies	Prob.	95% C.B.	Prob.	95% C.B.	Prob.	95% C.B.	Prob.	95% C.B.
State	0.226	[0.022;0.792]	0.123	[0.007;0.744]	0.040	[0.006;0.234]	0.033	[0.010;0.104]
District	0.226	[0.076;0.510]	0.123	[0.045;0.297]	0.040	[0.009;0.163]	0.033	[0.012;0.088]
Cluster	0.226	[0.048;0.629]	0.123	[0.018;0.514]	0.040	[0.004;0.286]	0.033	[0.004;0.225]

Predicted probability from four-level logistic regression: 95% C.B – 95% coverage bounds. Probability for 2006 (NFHS-3) was not estimated due to non-availability of district identifiers in the data.

© 2023 Rajpal S et al. *JAMA Network Open.*
