# Supplementary material for: Patterns in the Prevalence of Unvaccinated Children Across 36 States and Union Territories in India, 1993-2021
Source: JAMA Netw Open. 2023 Feb 10;6(2):e2254919. doi: 10.1001/jamanetworkopen.2022.54919 (PMC9918883; doi:10.1001/jamanetworkopen.2022.54919)
Supplement: Supplement 2. — Data Sharing Statement [file jamanetwopen-e2254919-s002.pdf]

Data Sharing Statement

Rajpal. Patterns in the Prevalence of Unvaccinated Children Across 36 States and Union Territories in India, 1993-2021. *JAMA Netw Open*. Published February 10, 2023. doi:10.1001/jamanetworkopen.2022.54919

Data
Data available: No

Additional Information
Explanation for why data not available: This project used publicly accessible secondary data obtained from the DHS website. DHS data are available at https://dhsprogram.com (requiring a simple application).
